# Long-term effects of meteorological factors on severe fever with thrombocytopenia syndrome incidence in eastern China from 2014 to 2020: An ecological time-series study

**DOI:** 10.1371/journal.pntd.0012266

**Published:** 2024-06-25

**Authors:** Yao Wang, Xueying Tian, Bo Pang, Wei Ma, Zengqiang Kou, Hongling Wen

**Affiliations:** 1 Department of Epidemiology, School of Public Health, Cheeloo College of Medicine, Shandong University, Jinan, China; 2 Department of Microbiological Laboratory Technology, School of Public Health, Cheeloo College of Medicine, Shandong University, Jinan, China; 3 Infection Disease Control of Institute, Shandong Center for Disease Control and Prevention, Shandong Provincial Key Laboratory of Infectious Disease Prevention and Control, Jinan, China; University of California San Diego, UNITED STATES

## Abstract

**Background:**

Severe fever with thrombocytopenia syndrome (SFTS) is an emerging tick-borne disease with susceptibility influenced by meteorological factors. However, there is limited understanding of the delayed and interactive impacts of meteorological factors on SFTS incidence.

**Methods:**

Daily incidence data of SFTS and corresponding meteorological factors for the Jiaodong Peninsula in northeast China were collected from January 1, 2014, to December 31, 2020. Random forest regression model, based on custom search, was performed to compare the importance of meteorological factors. Generalized additive model with quasi-Poisson regression was conducted to examine the nonlinear relationships and interactive effects using penalized spline methods. A distributed lag nonlinear model with quasi-Poisson regression was constructed to estimate exposure-lag effects of meteorological factors.

**Results:**

The most important meteorological factor was weekly mean lowest temperature. The relationship between meteorological factors and SFTS incidence revealed a nonlinear and intricate pattern. Interaction analyses showed that prolonged sunshine duration posed a climatic risk within a specific temperature range for SFTS incidence. The maximum relative risk (RR) observed under extremely low temperature (-4°C) was 1.33 at lag of 15 week, while under extremely high temperature (25°C), the minimum RR was 0.65 at lag of 13 week. The RRs associated with both extremely high and low sunshine duration escalated with an increase in lag weeks.

**Conclusions:**

This study underscores that meteorological factors exert nonlinear, delayed, and interactive effects on SFTS incidence. These findings highlight the importance of understanding the dependency of SFTS incidence on meteorological factors in particular climates.

## Author summary

Severe fever with thrombocytopenia syndrome (SFTS), listed as a priority disease by World Health Organization in 2018, is an emerging tick-borne disease that poses a significant public health risk. As a climate-sensitive infectious disease, climatic variations have profound impacts on SFTS incidence. Previous studies have demonstrated the influence of meteorological variables on SFTS incidence, while few have focused on the delayed and interaction effects of these factors on SFTS incidence. To the best of our knowledge, this is the first study to investigate the interaction effects of meteorological factors on SFTS incidence, as well as the first study to estimate the relationship between meteorological factors and SFTS incidence in a humid subtropical climate region. In this study, we compared the relative importance of different meteorological factors on SFTS incidence and further explored the nonlinear trend, interaction effect, and exposure-lag effect of meteorological factors on SFTS incidence. Our results highlight the importance of understanding the dependency of SFTS incidence on meteorological factors in particular climates and may inform resource allocation plans for disease control in scenarios of climate change.

## Introduction

Severe fever with thrombocytopenia syndrome (SFTS) is an emerging tick-borne infectious disease characterized by fever, thrombocytopenia, gastrointestinal symptoms, and neurological symptoms, caused by SFTS virus (SFTSV) [1]. SFTS was first identified in China in 2009 [2], and has since been discovered in Japan [3], South Korea [4], and Vietnam [5]. In 2018, SFTS, posing a public health risk due to its high fatality rate, limited treatment measures, and absence of a preventive vaccine, was listed as a priority disease by WHO [6].

SFTSV is primarily transmitted to humans by the bite of infected ticks, direct contact with SFTS patient secretions or blood, and probable aerosol transmission [7,8], and *Haemaphysalis longicornis (H longicornis)* was considered as the main vector of SFTSV [9]. The spatial-temporal distribution and spread of SFTSV depend on complex interactions between multiple ecological, environmental, and societal variables. Environmental variables can potentially serve as proxies for tick habitat in predicting SFTS incidence, as they affect ticks, their hosts, and habitat [10]. Previous study has indicated that meteorological factors are independent risk determinants for the occurrence of SFTS in China [11]. However, the effects of meteorological factors on SFTS incidence vary in form and by region due to geographical and climatic differences. Meteorological variables can affect the ecology of SFTSV by influencing tick-human interactions, tick growth dynamics and virus replication [12]. Some studies have identified the potential high-risk areas for SFTS incidence based on multiple meteorological factors, with temperature, precipitation, and sunshine duration playing important roles [13–15]. Previous studies has also observed complex and debatable nonlinear relationship between meteorological factors and SFST incidence, where different meteorological factors have diverse suitable ranges contributing to SFTS incidence [16–20]. Though nonlinear effect of meteorological factors on SFTS incidence were explored, little is known about the interaction and exposure-lag effects of these factors.

Our study employed random forest regression model, generalized additive model (GAM) [21] and distributed lag nonlinear model (DLNM) [22] to compare the importance, estimate nonlinear trend and interaction effect, and exposure-lag effects of meteorological factors on SFST incidence based on surveillance data from the Jiaodong Peninsula, China. The Jiaodong Peninsula, situated between the humid subtropical and humid continental zones, has dry winter compared to other endemic regions with a fully humid climate [23,24]. The unique geographical and climatic environments may significantly influence the distribution and density of SFTS vectors and SFTS incidence. Our study may further inform scenario plans for climate-driven transmission control, and contribute to upgurading local health care services and policy action.

## Materials and methods

### Ethics statement

This study was approved by the Ethical Review Committee of the Shandong Center for Disease Control and Prevention (2021–47). The study did not involve human participants. All the methods employed in the study were by the applicable guidelines and regulations.

### Study area

**Fig 1:**
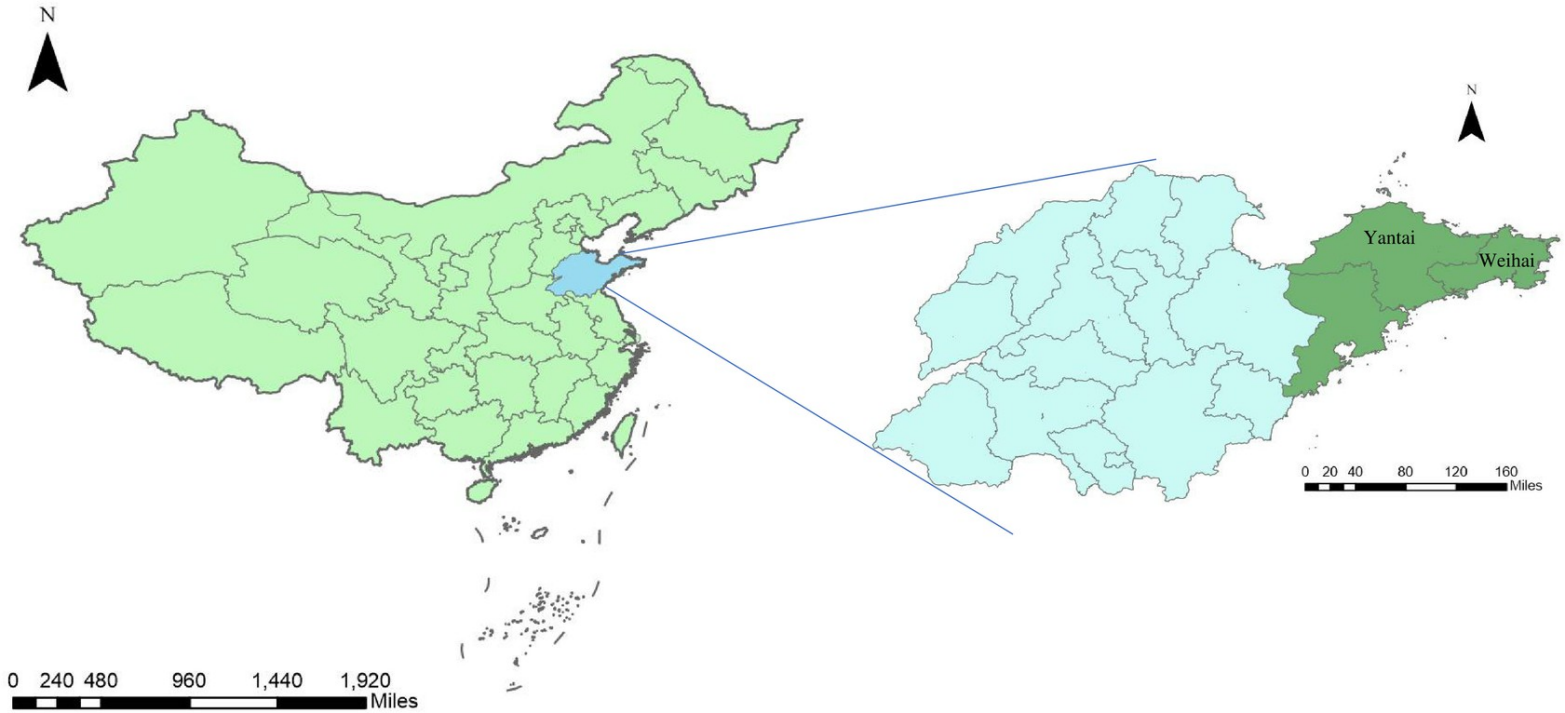
Location of the study area in China. The left figure depicts a map of China, with the blue-shaded area representing Shandong Province. The right figure illustrates the location of Jiaodong Peninsula within Shandong Province. The base layer of the map is from Resource and Environment Science and Data Center (https://www.resdc.cn/DOI/DOI.aspx?DOIID=120).

Jiaodong Peninsula is located in east Shandong Province between longitudes 119°30′ E—122°42′ E and latitudes 35°35′ N—38°23′ N (Fig 1). According to the Koeppen-Geiger climate classification, Jiaodong Peninsula, spanning the transition between humid subtropical and humid continental zones, belongs to a warm temperature with dry winter and hot summer [23].

### Data collection

According to Chinese law, SFTS cases must be reported to the China Information System for Diseases Control and Prevention (CISDCP) within 24 hours of diagnosis, as stipulated in the National Guideline for Prevention and Control of SFTS issued by the National Health Commission. Weekly data on laboratory-diagnosed SFTS patients in endemic areas were collected from the CISDCP from January 1, 2014, to December 31, 2020. Meteorological data over the same period, including mean temperature (°C), highest temperature (°C), lowest temperature (°C), air pressure (hPa), precipitation (mm), relative humidity (%), mean wind speed (m/s), mean speed of gustiness (m/s), maximum speed of gustiness (m/s), and sunshine duration (h), were collected from the China Meteorological Data Sharing Service System (http://data.cma.cn/).

### Data analysis

A descriptive analysis was initially conducted to describe the temporal trend of SFTS cases and meteorological factors during the study period in Jiaodong Peninsula. A random forest regression model was performed to assess the importance of meteorological factors on SFTS incidence. The random forest model is widely used for data prediction and interpretation. In this study, custom search algorithms were utilized to optimize tuning parameters for the random forest model. The increase in the mean of squared residuals (%IncMSE) was used to measure the variable importance. Spearman correlation analysis was performed to evaluate the relationship among meteorological factors. To ensure the stability of the random forest regression model results, three sensitivity analysis methods were employed. Firstly, the random forest regression model was repeated 10 times, and the average of variable relative importance scores was calculated. Secondly, after removing variables with high correlation but minimal importance, the random forest regression was re-executed. Thirdly, the recursive feature elimination (RFE) method was utilized to investigate variable importance [25]. Variables were selected for further investigation by combining the significance test results (*P* < 0.05) of the importance scores derived from the random forest regression with the correlation analysis results. Specifically, if two variables showed high correlation (*r* > 0.7) [26], the one with a higher importance score was retained to avoid collinearity.

In this study, a log-linear GAM was used to analyze the association between meteorological factors and SFTS cases. We assumed that the weekly SFTS cases approximately followed a quasi-Poisson distribution, and constructed a model between the logarithm of expected SFTS cases and meteorological variables through a GAM with a Gaussian distribution family. The optimal degrees of freedom (df) for the spline function were estimated using generalized cross-validation criteria. We calibrated the model by incorporating temporal trends as a confounder. The model was defined as follows:

$$logY_{t}=\alpha +s({MF}_{t},df)+s(Week,df)$$

Where log (Y_t_) is the log-transformed of the number of SFTS cases on week_t_. α represents the intercept. MF_t_ is the meteorological factor on week_t_. s() refers to a thin plate spline function, which is based on the penalized smoothing spline. Week represents the number of weeks appearing SFTS cases. df is the degree of freedom.

Previous studies have demonstrated that meteorological variables influence infectious diseases has a lag effect [15,17,18]. However, GAM only considers the impact within a particular period, leading to high collinearity when exposure levels of several consecutive weeks are introduced without considering the lag distribution characteristics. DLNM combines GAM with a distributed lag linear model, adding a lag dimension to the exposure-response relation though the cross-basis function, and simultaneously evaluates the lag effect and nonlinear effect. The model can be written as follows:

$$logE(Y_{t})=\alpha +cb(M,{df}_{1})+ns({Cov}_{t},{df}_{2})+ns(Week,{df}_{3})$$

where E(Yt) represents the number of weekly reported SFTS cases on week t; α is the intercept; cb means the cross-basis matrix function of meteorological variable; week is a time variable used to control for seasonal and long-term trends; ns represents the nature spline function. To control for confounding factors, the meteorological factors not included in function were set as covariate variables (Cov). Considering the life cycle of tick and the incubation period, we applied the model with a varying lags period of up to 27 weeks [2,15].

We also performed several sensitivity analyses. First, we varied the df to 5 and 8 for meteorological factors in single-variable GAM analysis. Second, in multiple-variables GAM analysis, each meteorological factor was adjusted by extra variables with correlation coefficient < 0.7. For the DLNM analysis, we initially included only lowest temperature and sunshine duration in the model. When one factor was included in the function, the other one was set as covariate variable. Then, we adjusted the df for relative humidity (4 and 5) and maximum speed of gustiness (4 and 5) to assess the robustness.

All analyses were performed using the “caret”, “randomForest”, “rfPermute”, “corrplot”, “dlnm” and “mgcv” packages in R software (version 4.1.0). The confidence interval (CI) of all two-side statistical tests was set as 95%, and *P* < 0.05 was considered statistically significant.

## Results

### Summary of meteorological factors and SFTS cases

During the study period, 1,621 SFTS cases were reported in Weihai and Yantai. Weekly averages for SFTS cases, lowest temperature, mean temperature, highest temperature, air pressure, sunshine duration, wind speed, mean speed of gustiness, maximum speed of gustiness, relative humidity, and precipitation were 4.5 cases, 10.3°C, 13.6°C, 17.4°C, 1009.9 hpa, 4.4 h, 3.3 m/s, 6.6 m/s, 10.2 m/s, 66.6%, and 1.5 mm, respectively (S1 Table). S1 Fig illustrates temporal trends of SFTS cases and meteorological factors, indicating a potential association between meteorological factors and SFTS incidence with cases concentrated from May to October (S2 Fig).

### Relationship between meteorological factors and SFTS incidence

**Fig 2:**
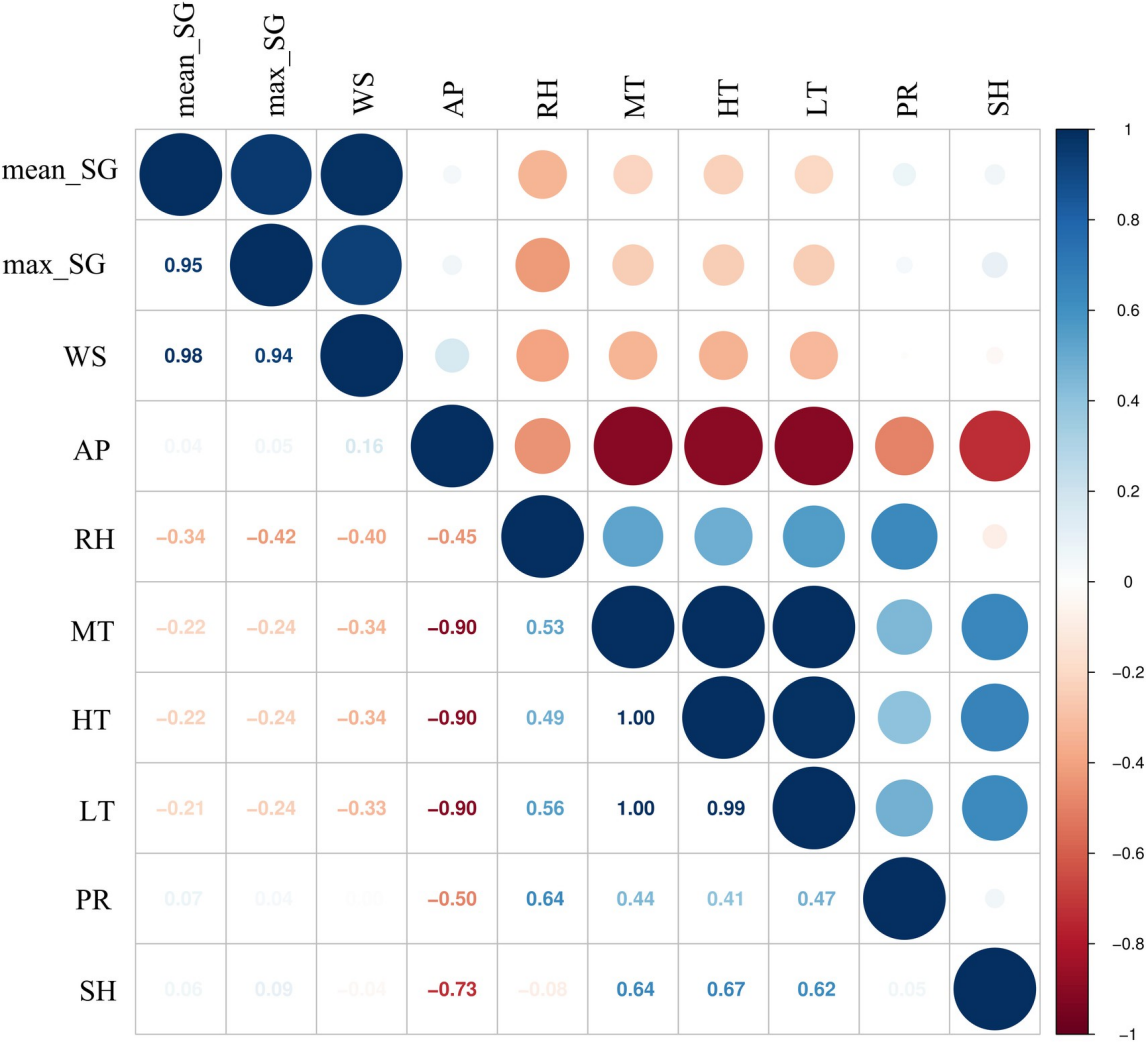
Spearman correlation coefficients between meteorological factors and weekly incidence of SFTS. The deeper the shade of blue or red, the larger the circle, indicating a stronger correlation between variables. mean_SG: weekly mean speed of gustiness; max_SG: weekly mean maximum speed of gustiness; WS: weekly mean wind speed; AP: weekly mean air pressure; RH: weekly mean relative humidity; MT: weekly mean temperature; HT: weekly mean highest temperature; LT: weekly mean lowest temperature; PR: weekly mean precipitation; SH: weekly mean sunshine duration.

Spearman correlation analysis indicated that air pressure, wind speed, mean speed of gustiness, and maximum speed of gustiness were negatively correlated with the number of weekly SFTS cases (Fig 2). In contrast, lowest temperature, mean temperature, highest temperature, sunshine duration, relative humidity, and precipitation were positively correlated with the incidence of SFTS (Fig 2).

**Fig 3:**
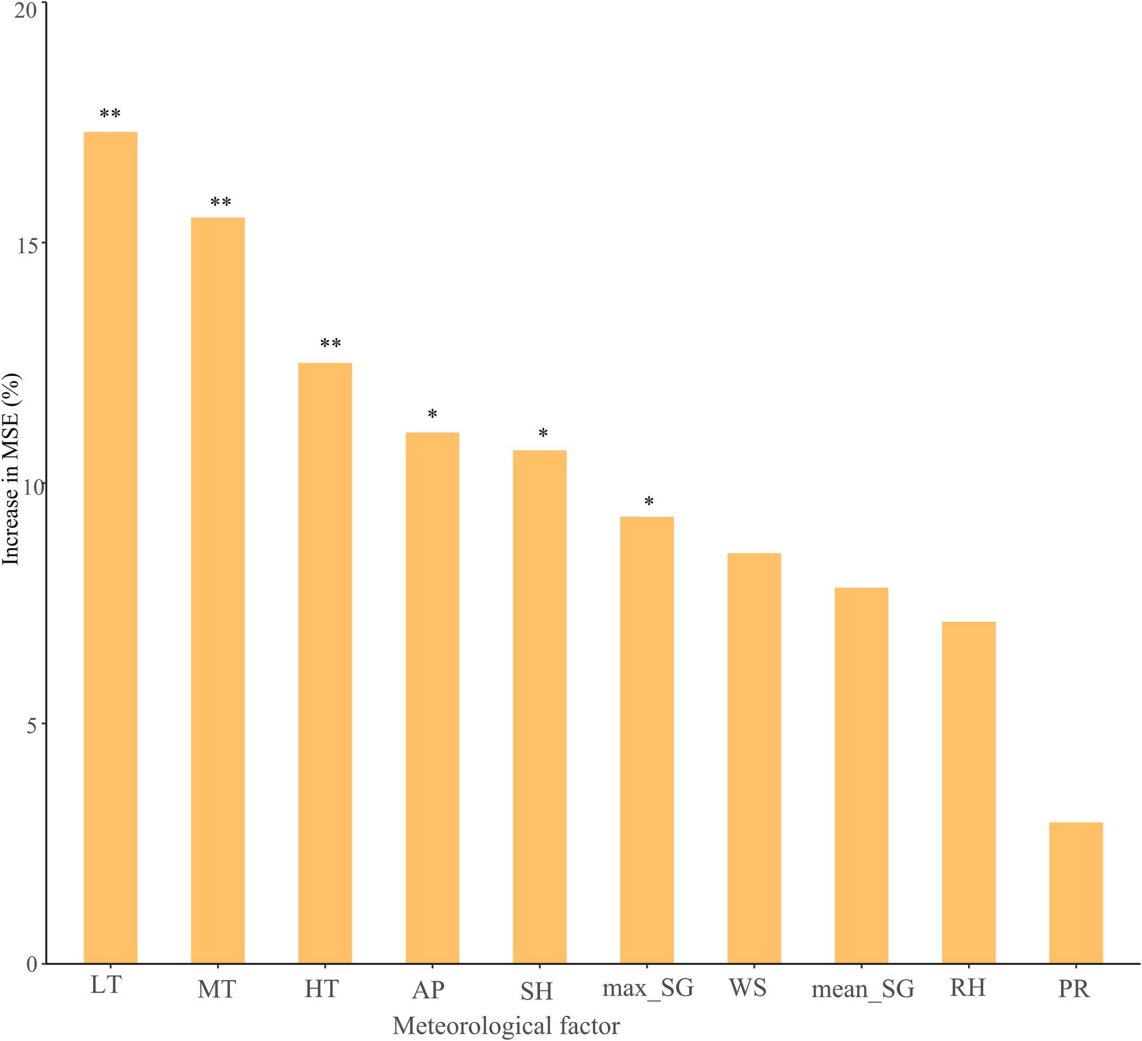
Importance of the meteorological factors on SFTS incidence. %IncMSE means the increase in the means of squared residuals. The predictive variable importance scores were computed using a random forest model. The significance of variable importance scores was assessed through a permutation test, which provided information on the statistical significance of the variable importance. mean_SG: weekly mean speed of gustiness; max_SG: weekly mean maximum speed of gustiness; WS: weekly mean wind speed; AP: weekly mean air pressure; RH: weekly mean relative humidity; MT: weekly mean temperature; HT: weekly mean highest temperature; LT: weekly mean lowest temperature; PR: weekly mean precipitation; SH: weekly mean sunshine duration. MSE: mean of squared residuals. ** *P* < 0.01; * *P* < 0.05.

Random forest regression analysis suggested that the most important meteorological factor for SFTS was lowest temperature, with a %IncMSE greater than 15%, followed by mean temperature, highest temperature, air pressure and sunshine duration. The least important factor was precipitation, with an %IncMSE less than 5% (Fig 3). Sensitivity analysis indicated that the results of the random forest regression were robust (S3–S5 Figs).

Synthesizing the results of Spearman correlation and random forest analysis, the subsequent model included lowest temperature, sunshine duration, maximum speed of gustiness and relative humidity.

**Fig 4:**
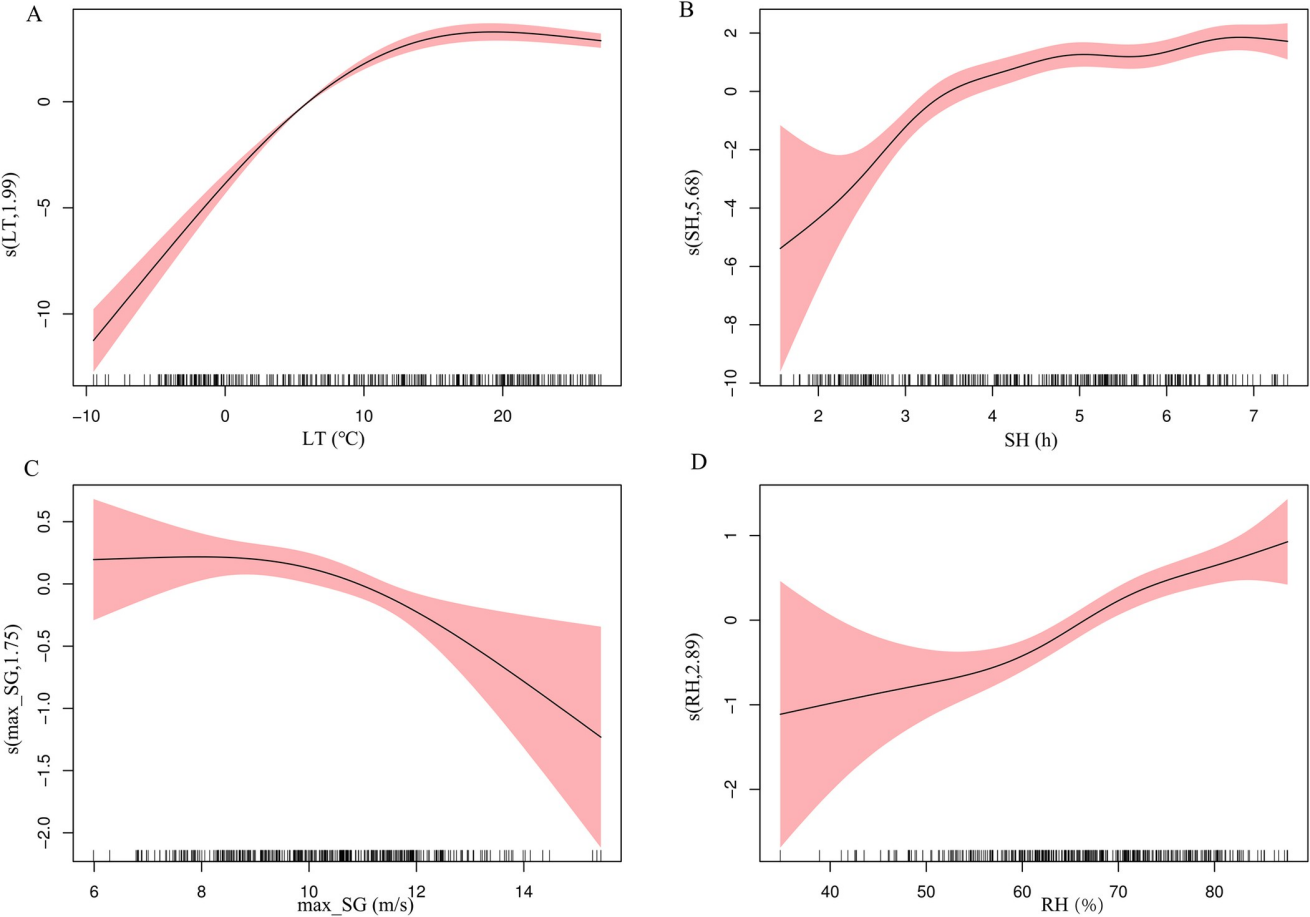
Exposure-response curves for the effects of meteorological factors on weekly SFTS cases in the single-variable model using univariate GAM (Fig 4A–4D indicate the exposure-response relationship between lowest temperature, sunshine duration, maximum speed of gustiness, relative humidity and SFTS cases, respectivaly). The x-axis is the meteorological parameters. The y-axis indicates the contribution of smoother to the fitted values. LT: weekly mean lowest temperature; SH: weekly mean sunshine duration; max_SG: weekly mean maximum speed of gustiness; RH: weekly mean relative humidity.

**Fig 5:**
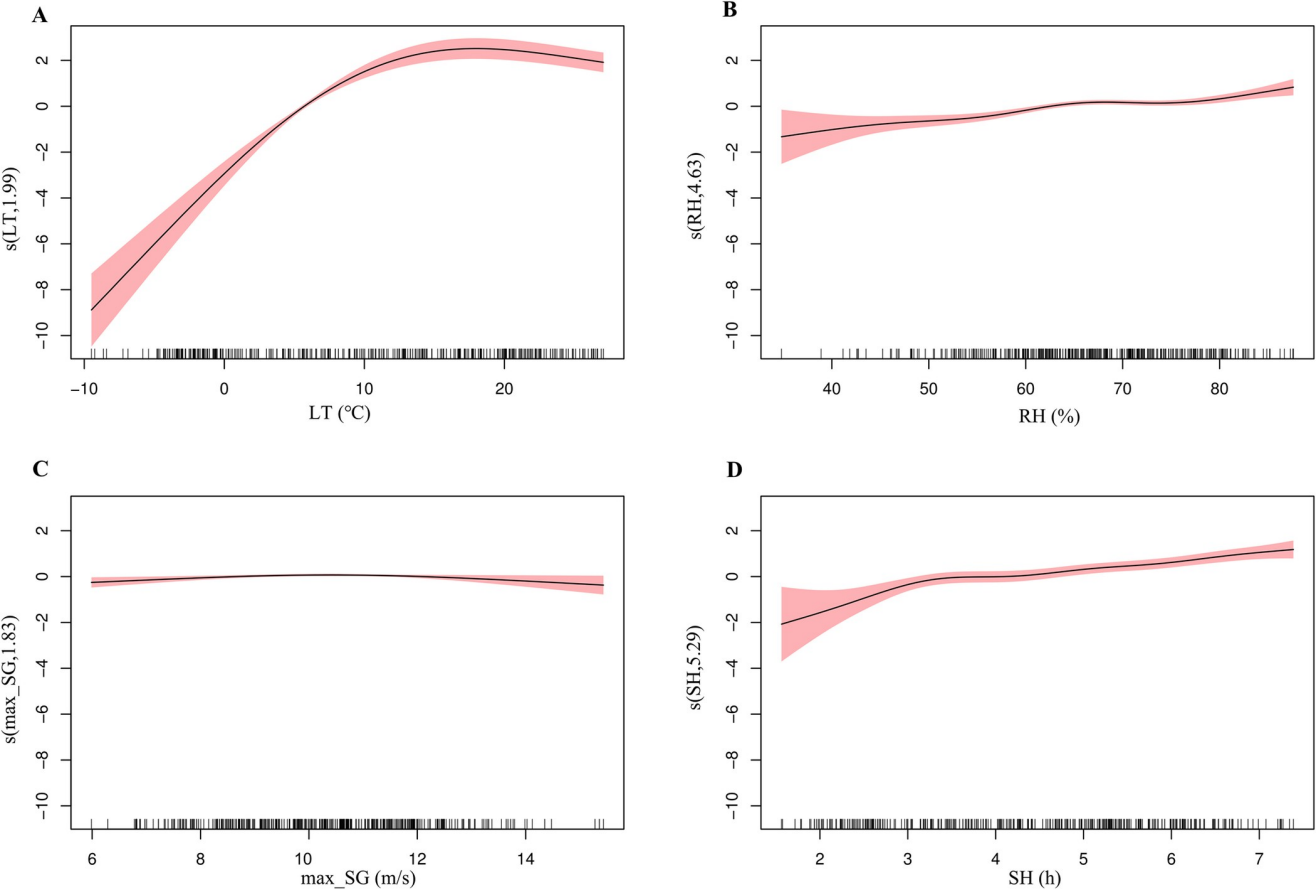
Exposure-response curves for the effects of meteorological factors on weekly SFTS cases in the multiple-variables model using multivariable GAM (Fig 5A–5D indicate the exposure-response relationship between lowest temperature, relative humidity, maximum speed of gustiness, sunshine duration and SFTS cases, respectivaly). The x-axis is the meteorological parameters. The y-axis indicates the contribution of the smoother to the fitted values. LT: weekly mean lowest temperature; SH: weekly mean sunshine duration; max_SG: weekly mean maximum speed of gustiness; RH: weekly mean relative humidity.

**Fig 6:**
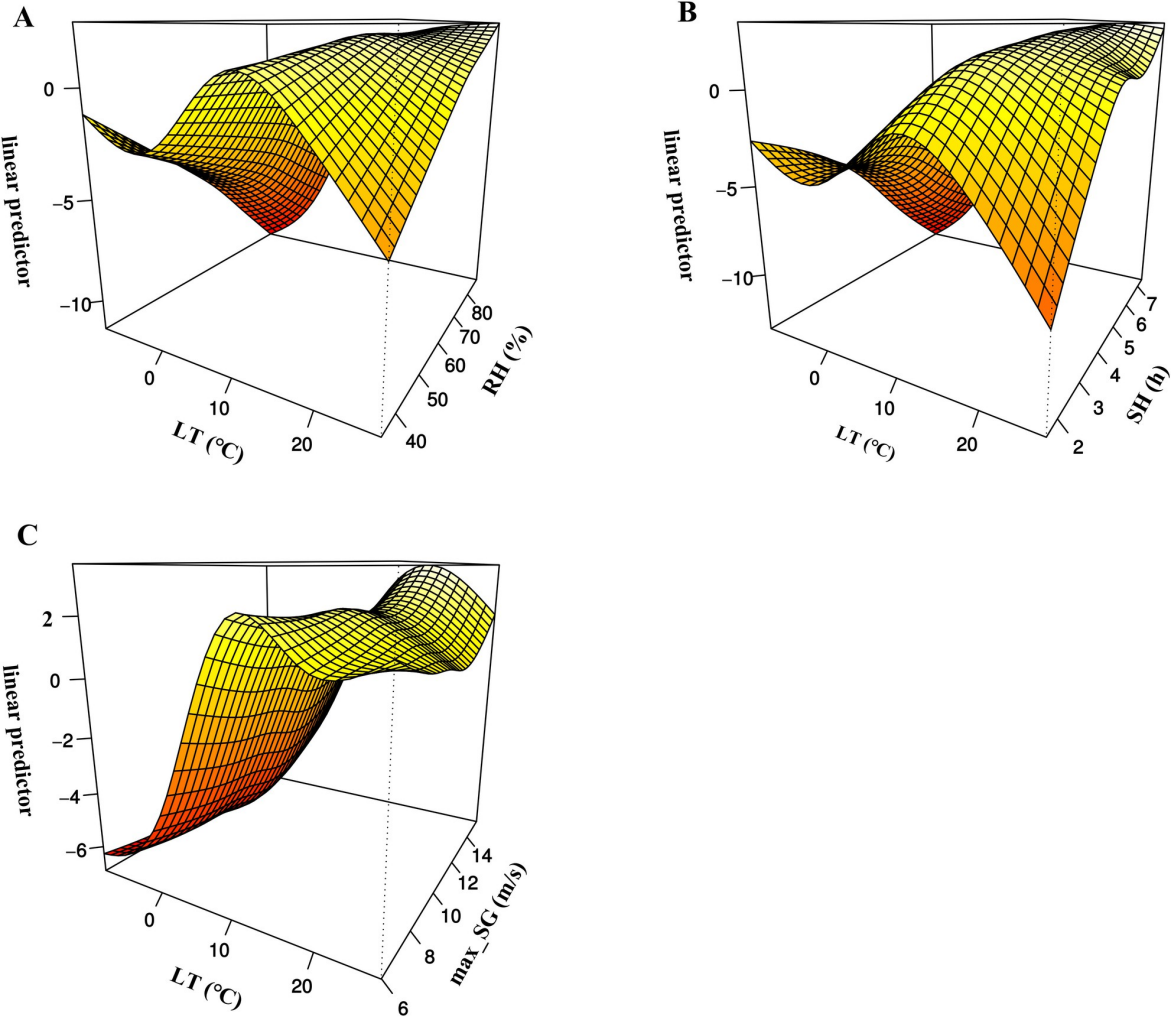
The interactive effects of meteorological factors and SFTS incidence in Jiaodong Peninsula. The interactive effects were estimated using GAM. Fig 6A shows the interactive effect between lowest temperature and sunshine duration; Fig 6B shows the interactive effect between lowest temperature and sunshine duration; Fig 6C shows the interactive effect between lowest temperature and maximum speed of gustiness. LT: weekly mean lowest temperature; SH: weekly mean sunshine duration; max_SG: weekly mean maximum speed of gustiness.

First, we used GAM to perform single-variable analyses for different meteorological factors (Fig 4). The risk of SFTS increased with weekly mean lowest temperature when it was below 20°C. Elevated sunshine duration and relative humidity contributed to SFTS incidence. We further used GAM to conduct multiple-variables on these four factors (Fig 5). Compared to lowest temperature, the effect of maximum speed of gustiness and relative humidity on SFTS incidence became negligible. We also evaluated the interaction effect of lowest temperature, sunshine duration, maximum speed of gustiness and relative humidity on SFTS incidence. There were significant interaction effects between lowest temperature and the other three factors (S2 Table). The interaction effect of these factors is shown in Fig 6. The interaction effect between sunshine duration, relative humidity and lowest temperature was complex. The interaction effects of relative humidity, sunshine duration and lowest temperature on SFTS incidence were similar. At a certain lowest temperature, different levels of relative humidity and sunshine duration had varied effects on SFTS incidence. Lowest temperature and maximum speed of gustiness presented an obvious interaction effect when the lowest temperature exceeded 10°C approximately.

**Fig 7:**
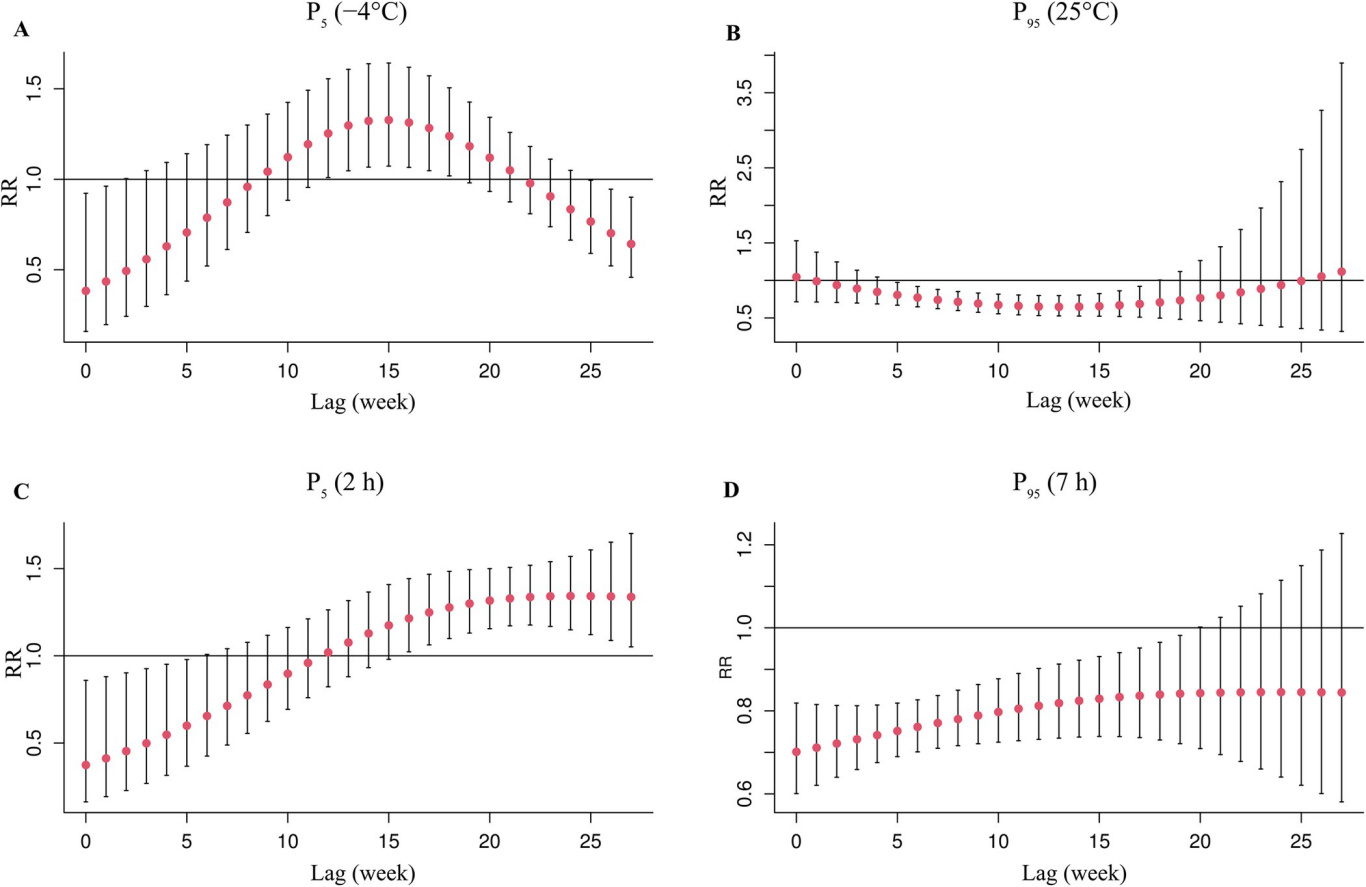
The lag effects between meteorological factors and SFTS incidence. Fig 7A and 7B displayed the effects of the 5th (-4°C) and 95th (25°C) percentiles of lowest temperature across different lag periods, compared to the median temperature, respectively; Fig 7C and 7D displayed the effects of the 5th (2h) and 95th (7h) percentiles of sunshine duration across different lag periods, compared to the median sunshine duration, respectively. LT: weekly mean lowest temperature; SH: weekly mean sunshine duration.

We further evaluated the lag and nonlinear effects of lowest temperature and sunshine duration using DLNM. We constructed 3-D and contour plots of the two factors to comprehensively summarize the exposure-lag-response association (S6 Fig). The reference level was set as the corresponding variable’s median value. Generally, when the lowest temperature was higher than 10.8°C, the relative risk of SFTS decreased with an increase in the lowest temperature. When the sunshine duration was higher than 4.6 hours, the risk of SFTS decreased as sunshine time increased. The RR with 95%CI of SFTS incidence among lag weeks was calculated with the 95th and 5th percentile of lowest temperature and sunshine duration (Fig 7). Under extremely low temperature (5th percentile of lowest temperature), the RRs for lag weeks 12–18 were significantly high, with the highest effect estimate occurring on the lag 15 week (RR = 1.33, 95%CI: 1.07–1.64). Furthermore, under extremely high temperature (95th percentile of lowest temperature), the RRs for lag weeks 5–17 were significantly low, with the lowest effect estimate occurring on the lag 13 week (RR = 0.65, 95%CI: 0.53–0.80). In the sunshine duration slice plots, the RRs were significantly high from lag 16 week (RR = 1.21, 95%CI: 1.02–1.44) under extremely low sunshine duration; however, the RRs before lag 19 week (RR = 0.84, 95%CI: 0.72–0.98) were significantly low.

**Fig 8:**
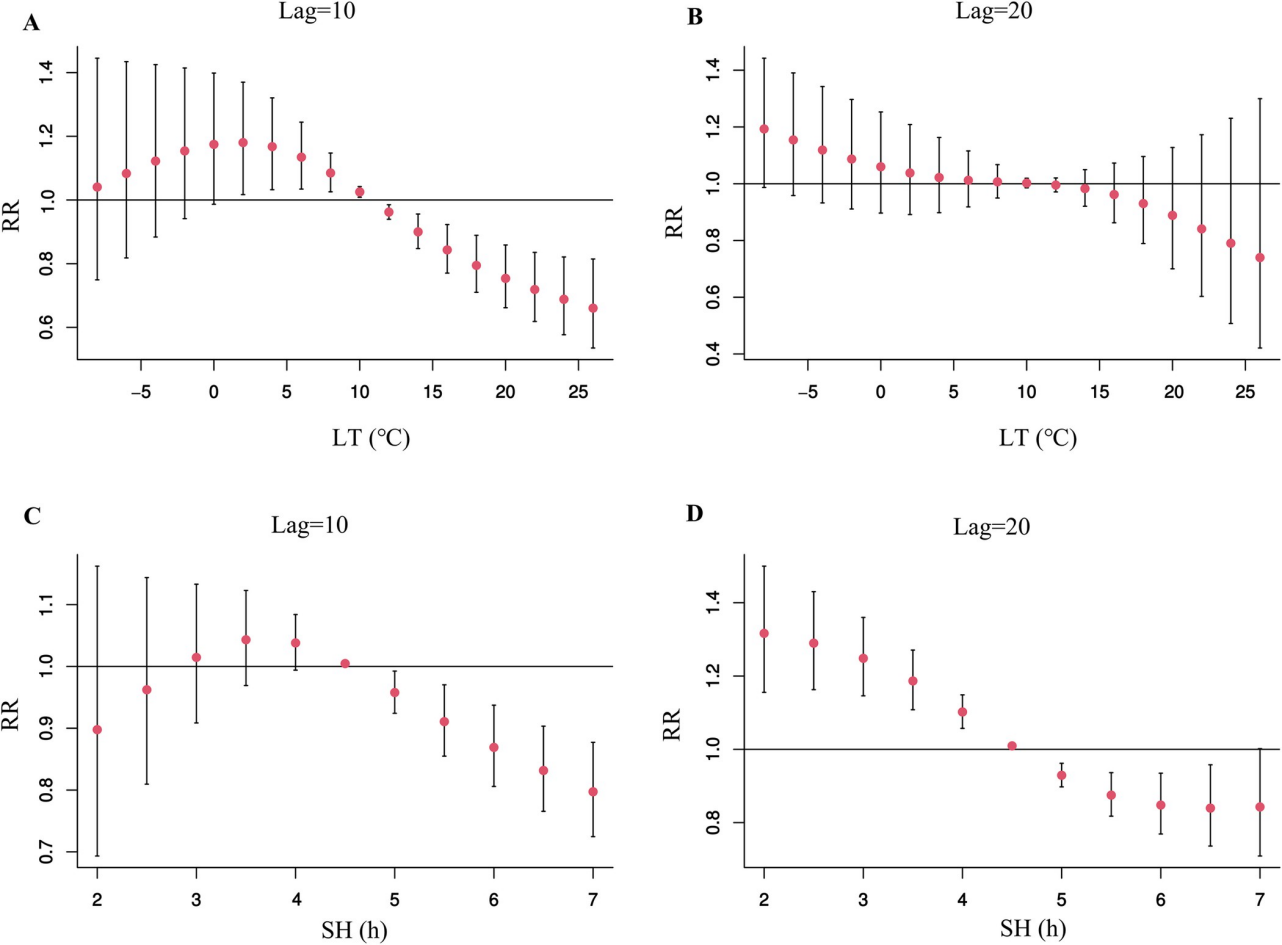
The lag-specific effects of meteorological factors on SFTS incidence. Fig 8A and 8B represent the effects of different minimum temperatures at the lag 10th and 20th weeks, respectively; Fig 8C and 8D represent the effects of different sunshine durations at the lag 10th and 20th weeks, respectively. LT: weekly mean lowest temperature; SH: weekly mean sunshine duration.

Fig 8 shows the lag-specific association between different meteorological factors and SFTS incidence. For the lowest temperature, the RRs first increased and then decreased with increasing temperature values at lag 10 week, but decreased continuously at lag 20 week. No significant RR was observed at lag 20 week for the lowest temperature. The trend of sunshine duration was very similar to that of the lowest temperature. Significant RRs were observed when sunshine duration exceeded five hours at lag 10 week, with the lowest effect estimate (RR = 0.80, 95%CI: 0.72–0.88) corresponding to 7 hours.

The nonlinear trends and exposure-lag effects observed in the sensitivity analyses were consistent with those of our main model, indicating the robustness and reliability of our model (S7–S14 Figs).

## Discussion

In this study, the importance, delay, interaction effects of meteorological factors on SFTS incidence were investigated. Our study results show that lowest temperature, air pressure, sunshine duration and maximum speed of gustiness were related to SFTS incidence, with lowest temperature being the most important. Both lowest temperature and sunshine duration exhibit pronounced non-linear and delayed effects on the occurrence of SFTS. Moreover, lowest temperature and other meteorological factors collectively affect the risk of SFTS infection. To the best of our knowledge, this is the first study to investigate interaction effects of meteorological factors on SFTS incidence, and it is also the first study to estimate the relationship between meteorological factors and SFTS incidence in a humid subtropical climate region [23].

Previous studies have shown that temperature is one of the key environmental factors affecting SFTS occurrence with a complicated and nonlinear relationship [12,15,20,27]. Both low and high temperatures inhibit the meal-seeking activity of ticks, which can impact the geographic regions where the climate is suitable for tick activity and the diurnal duration of ticks’ activity [28]. A previous study showed that the suitable temperature range for SFTS occurrence was 11.6°C to 12.8°C [13]. In this study, we found that most SFST cases were identified during May-October, parallel to the period of tick activity [29]. During the growth cycle of ticks, larvae hibernate when the temperature is too low and become nymphs when the temperature reaches a certain level [30]. Yano et al. [18] found that the critical low temperatures for oviposition, egg hatching (developmental zero) and larval and nymphal moulting were 11.1°C, 12.2°C, 10.2°C and 11.8°C respectively. Another study reported that the RR increased with the increment of temperature and peak at 23°C, with high temperatures having acute and short-term effects [31]. This discrepancy may be attributed to different climatic and geographical environments. The Jiaodong Peninsula lies in the transition between the humid subtropical and humid continental zones with dry, long and cold winters. The lag trend of our study was consistent with the results of a previous study [31], but the estimated effect values were differential, possibly due to discrepancies in temperature indices and study areas.

The incidence of SFST was very sensitive and negatively related to air pressure. A possible explanation is that lower pressure may lead to a longer period of questing and a lower mortality rate in ticks [20], which could contribute to the high feeding success of nymphs on their natural hosts and increase the chances of human exposure to ticks. Although we did not identify an evident non-linear relationship between SFTS incidence and relative humidity, consistent with a previous study conducted in Shandong Province [13]. relative humidity played important roles in the dynamics of SFTS in Zhejiang Province [20]. This difference may be explained by environmental differences between regions; compared to Zhejiang Province, the Jiaodong Peninsula has a relatively dry climate. This distinction motivates us to prioritize the local area rather than larger a spatial scale. As noted in previous research, wind speed can influence the growth and reproduction of ticks [20]. A negative correlation between SFTS incidence and maximum speed of gustiness was found in this study. Continuous wind may increase the dispersal of carbon dioxide, an attractant for Ixodid ticks, but hasten forest air replacement by dryer air, resulting in an increase in tick mortality [32]. This may be the reason that the incidence decreased gradually as the wind speed rose. Consistent with previous studies, our study found that rising sunshine duration increased the risk of SFTS incidence, but the importance score was relatively lower than temperature and air pressure [13,20]. Because the time series of temperature and sunshine duration are in phase, the effect of sunshine duration may be an indirect function of temperature. In addition, the lag effects of different meteorological factors on the SFTS incidence varied. Generally, the different lag periods reflected that the delayed effect of each meteorological factor may be associated with the transmission of the infection being affected by various factors, including people’s tendency to go out, seasonal variation in the rodent population, and the proliferation of the virus in the external environment [31,32].

The results of interaction analysis in our study demonstrated complex and comprehensive effects of meteorological factors on SFTS incidence. In the suitable temperature range, longer sunshine duration is a risk climate condition for the occurrence of SFTS, consistent with the living habits of ticks and humans [30]. However, further studies are needed to explore the potential mechanisms of this interaction effect.

This study has several strengths. Firstly, this is the first study to explore the interaction effect of meteorological factors on SFTS incidence in a humid subtropical climate region. Secondly, strict quality control for data ensured the credibility of this research. All SFTS cases included in our study underwent laboratory confirmation, eliminating the potential for misclassification. Thirdly, our time series of SFTS infections was based on the date of disease onset, minimizing errors caused by delays in notification. Finally, the selection of lag weeks was based on the tick life cycle rather than the goodness-of-fit of the model, strengthening the biological plausibility of result interpretation.

Some limitations of our study should be noted. Firstly, the collection of SFTS cases depended on passive surveillance, potentially leading to underreporting due to limited medical facilities and detection capability. Fortunately, this situation has improved remarkably in recent years. Additionally, the sample size included in this study was sufficient for the models, and comprehensive sensitivity analyses robustly affirmed the stability of results. Secondly, some potential determinants of human SFTSV infections were not included in our models due to data limitations. These factors include, but are not limited to, tick density and the infection rate of SFTSV in ticks and animal hosts, which could bias risk evaluation. However, ticks, as seasonal organism, undergo reproduction and growth influenced by climate and meteorological conditions. Previous research indicates that the meteorological conditions conducive to tick growth closely resemble the seasonal environment in which SFTS is prevalent. Therefore, while tick data would enhance the value of the model results, the current model based on meteorological conditions remains meaningful in their absence. Despite the lack of tick data, the model captures relevant aspects of the ecological context that contribute to SFTS prevalence. Thirdly, the results of this study may not apply to other regions due to differences in climate and geographical conditions.

## Conclusion

Our study explored the important meteorological variables for SFTS incidence, and further analyzed the non-linear trend, exposure-lag-effect and interaction effect of meteorological factors on SFTS incidence, which provides information to better understand the effect of meteorological factors variation on SFTS incidence and may inform resource allocation plans for disease control in climate change scenarios.

## Supporting information

S1 Fig Time series plot of SFTS cases and meteorological factors. A: weekly mean air pressure; B: weekly mean temperature; C: weekly mean highest temperature; D: weekly mean lowest temperature; E: weekly mean precipitation; F: weekly mean relative humidity; G: weekly mean wind speed; H: weekly mean speed of gustiness; I: weekly mean maximum speed of gustiness; J: weekly mean sunshine duration; K: weekly total SFTS cases.
(TIF)

S2 Fig Monthly distribution of SFTS in Jiaodong Peninsula, China, 2016–2020. (TIF)

S3 Fig The average of variable importance scores from 10 repeated random forest regression models. %IncMSE means the increase in the means of squared residuals. mean_SG: weekly mean speed of gustiness; max_SG: weekly mean maximum speed of gustiness; WS: weekly mean wind speed; AP: weekly mean air pressure; RH: weekly mean relative humidity; MT: weekly mean temperature; HT: weekly mean highest temperature; LT: weekly mean lowest temperature; PR: weekly mean precipitation; SH: weekly mean sunshine duration. MSE: mean of squared residuals.
(TIF)

S4 Fig Random forest regression model without the precipitation factor. %IncMSE means the increase in the means of squared residuals. mean_SG: weekly mean speed of gustiness; max_SG: weekly mean maximum speed of gustiness; WS: weekly mean wind speed; AP: weekly mean air pressure; RH: weekly mean relative humidity; MT: weekly mean temperature; HT: weekly mean highest temperature; LT: weekly mean lowest temperature; SH: weekly mean sunshine duration. MSE: mean of squared residuals.
(TIF)

S5 Fig Random forest regression model based on the recursive feature elimination. mean_SG: weekly mean speed of gustiness; max_SG: weekly mean maximum speed of gustiness; WS: weekly mean wind speed; AP: weekly mean air pressure; RH: weekly mean relative humidity; MT: weekly mean temperature; HT: weekly mean highest temperature; LT: weekly mean lowest temperature; PR: weekly mean precipitation; SH: weekly mean sunshine duration.
(TIF)

S6 Fig The 3-D graphs and contour plots of meteorological factors. S3A and S3C Fig are 3-D graphs, S3B and S3D Fig are contour plots; S3A and S3B Fig show the effects corresponding to different combinations of lowest temperatures and lag weeks; S3C and S3D show Fig the effects corresponding to different combinations of sunshine durations and lag weeks; LT: weekly mean lowest temperature; SH: weekly mean sunshine duration.
(TIF)

S7 Fig Sensitivity analysis for single-variable generalized additive model. S4A and S4B Fig illustrate the exposure-curve relationship between lowest temperature and SFTS incidence for degrees of freedom 5 and 8, respectively; S4C and S4D Fig illustrate the exposure-curve relationship between relative humidity and SFTS incidence for degrees of freedom 5 and 8, respectively; S4E and S4F Fig illustrate the exposure-curve relationship between maximum speed of gustiness and SFTS incidence for degrees of freedom 5 and 8, respectively; S4G and S4H Fig illustrate the exposure-curve relationship between sunshine duration and SFTS incidence for degrees of freedom 5 and 8, respectively; LT: weekly mean lowest temperature; SH: weekly mean sunshine duration; max_SG: weekly mean maximum speed of gustiness; RH: weekly mean relative humidity.
(TIF)

S8 Fig Sensitivity analysis for multiple-variable generalized additive model. S5A Fig: adjusted for relative humidity, precipitation, wind speed and sunshine duration; S5B Fig: adjusted for lowest temperature, precipitation, wind speed and sunshine duration; S5C Fig: adjusted for lowest temperature, precipitation, relative humidity and sunshine duration; S5D Fig: adjusted for lowest temperature, precipitation, relative humidity and wind speed. LT: weekly mean lowest temperature; SH: weekly mean sunshine duration; max_SG: weekly mean maximum speed of gustiness; RH: weekly mean relative humidity.
(TIF)

S9 Fig Sensitivity analysis for lag effect between meteorological factors and SFTS incidence after removing maximum speed of gustiness and relative humidity. The upper-left and upper-right graph represent the effects of the 5th (-4°C) and 95th (25°C) percentiles of lowest temperature across different lag periods, compared to the median temperature, respectively; the lower-left and lower-right graph represent the effects of the 5th (2h) and 95th (7h) percentiles of sunshine duration across different lag periods, compared to the median sunshine duration, respectively. LT: weekly mean lowest temperature; SH: weekly mean sunshine duration.
(TIF)

S10 Fig Sensitivity analysis for lag-specific effect of meteorological factors on SFTS incidence after removing maximum speed of gustiness and relative humidity. The upper-left and upper-right graph represent the effects of different minimum temperatures at the lag 10th and 20th weeks, respectively; the lower-left and lower-right graph represent the effects of different sunshine durations at the lag 10th and 20th weeks, respectively. LT: weekly mean lowest temperature; SH: weekly mean sunshine duration.
(TIF)

S11 Fig Sensitivity analysis for lag effect between meteorological factors and SFTS incidence by setting degree of freedom to 4. The upper-left and upper-right graph represent the effects of the 5th (-4°C) and 95th (25°C) percentiles of lowest temperature across different lag periods, compared to the median temperature, respectively; the lower-left and lower-right graph represent the effects of the 5th (2h) and 95th (7h) percentiles of sunshine duration across different lag periods, compared to the median sunshine duration, respectively. LT: weekly mean lowest temperature; SH: weekly mean sunshine duration.
(TIF)

S12 Fig Sensitivity analysis for lag-specific effect of meteorological factors on SFTS incidence by setting degree of freedom to 4. The upper-left and upper-right graph represent the effects of different minimum temperatures at the lag 10th and 20th weeks, respectively; the lower-left and lower-right graph represent the effects of different sunshine durations at the lag 10th and 20th weeks, respectively; LT: weekly mean lowest temperature; SH: weekly mean sunshine duration.
(TIF)

S13 Fig Sensitivity analysis for lag effect between meteorological factors and SFTS incidence by setting degree of freedom to 5. The upper-left and upper-right graph represent the effects of the 5th (-4°C) and 95th (25°C) percentiles of lowest temperature across different lag periods, compared to the median temperature, respectively; the lower-left and lower-right graph represent the effects of the 5th (2h) and 95th (7h) percentiles of sunshine duration across different lag periods, compared to the median sunshine duration, respectively; LT: weekly mean lowest temperature; SH: weekly mean sunshine duration.
(TIF)

S14 Fig Sensitivity analysis for lag-specific effect of meteorological factors on SFTS incidence by setting degree of freedom to 5. The upper-left and upper-right graph represent the effects of different minimum temperatures at the lag 10th and 20th weeks, respectively; the lower-left and lower-right graph represent the effects of different sunshine durations at the lag 10th and 20th weeks, respectively; LT: weekly mean lowest temperature; SH: weekly mean sunshine duration.
(TIF)

S1 Table Descriptive statistics of weekly SFTS cases and meteorological factors. (XLSX)

S2 Table Model test of the interaction analysis using GAM. LT: weekly mean lowest temperature; SH: weekly mean sunshine duration; max_SG: weekly mean maximum speed of gustiness; RH: weekly mean relative humidity; Edf: effective degrees of freedom; Ref.df: reference degrees of freedom; F-value is the value of variables using F test.
(XLSX)
