# Supplementary figures and images for: Long-term effects of meteorological factors on severe fever with thrombocytopenia syndrome incidence in eastern China from 2014 to 2020: An ecological time-series study
Source: PLoS Negl Trop Dis. 2024 Jun 25;18(6):e0012266. doi: 10.1371/journal.pntd.0012266 (PMC11230590; doi:10.1371/journal.pntd.0012266)

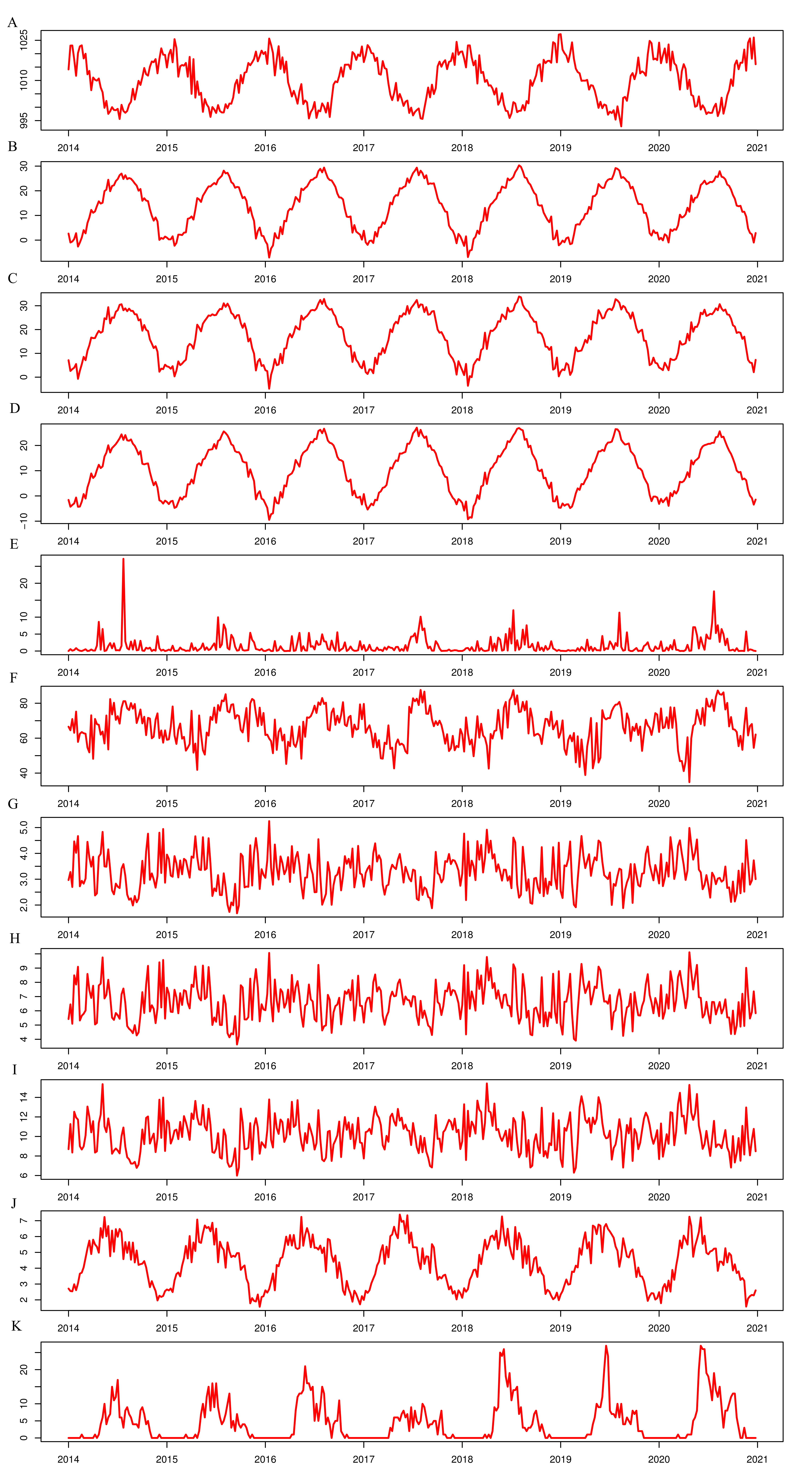

Supplement: S1 Fig — A: weekly mean air pressure; B: weekly mean temperature; C: weekly mean highest temperature; D: weekly mean lowest temperature; E: weekly mean precipitation; F: weekly mean relative humidity; G: weekly mean wind speed; H: weekly mean speed of gustiness; I: weekly mean maximum speed of gustiness; J: weekly mean sunshine duration; K: weekly total SFTS cases. (TIF) [file pntd.0012266.s001.tif]

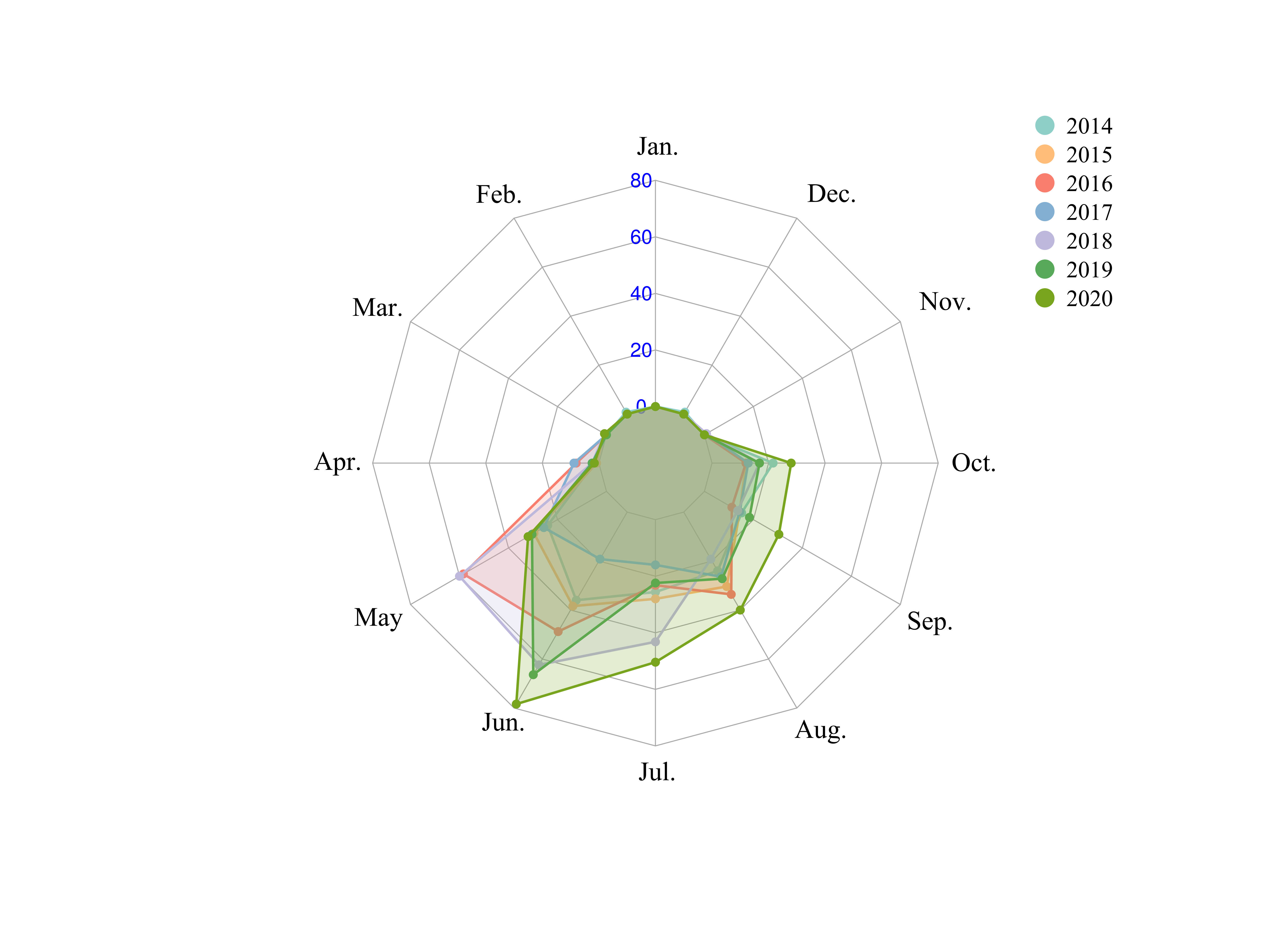

Supplement: S2 Fig — (TIF) [file pntd.0012266.s002.tif]

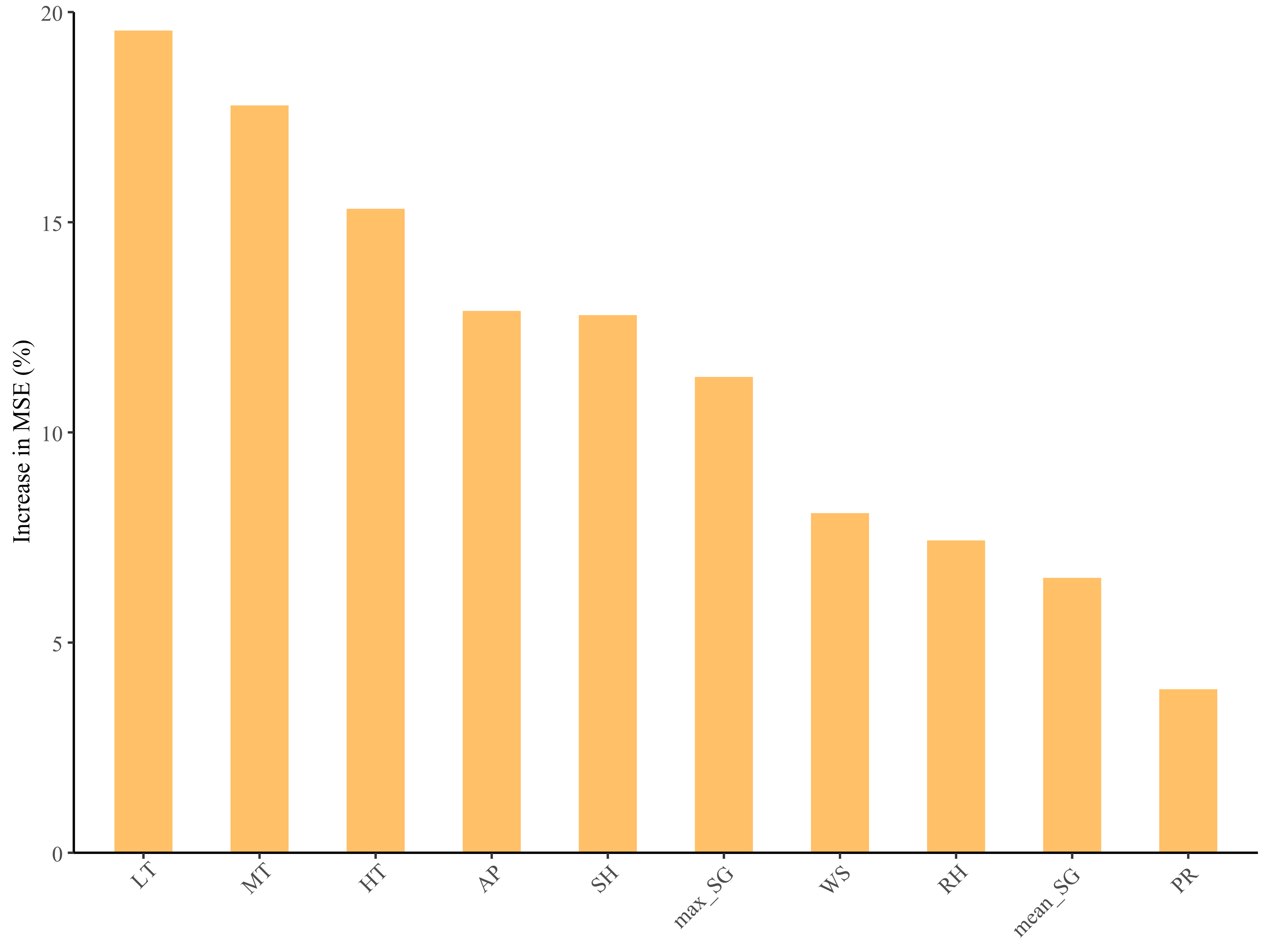

Supplement: S3 Fig — %IncMSE means the increase in the means of squared residuals. mean_SG: weekly mean speed of gustiness; max_SG: weekly mean maximum speed of gustiness; WS: weekly mean wind speed; AP: weekly mean air pressure; RH: weekly mean relative humidity; MT: weekly mean temperature; HT: weekly mean highest temperature; LT: weekly mean lowest temperature; PR: weekly mean precipitation; SH: weekly mean sunshine duration. MSE: mean of squared residuals. (TIF) [file pntd.0012266.s003.tif]

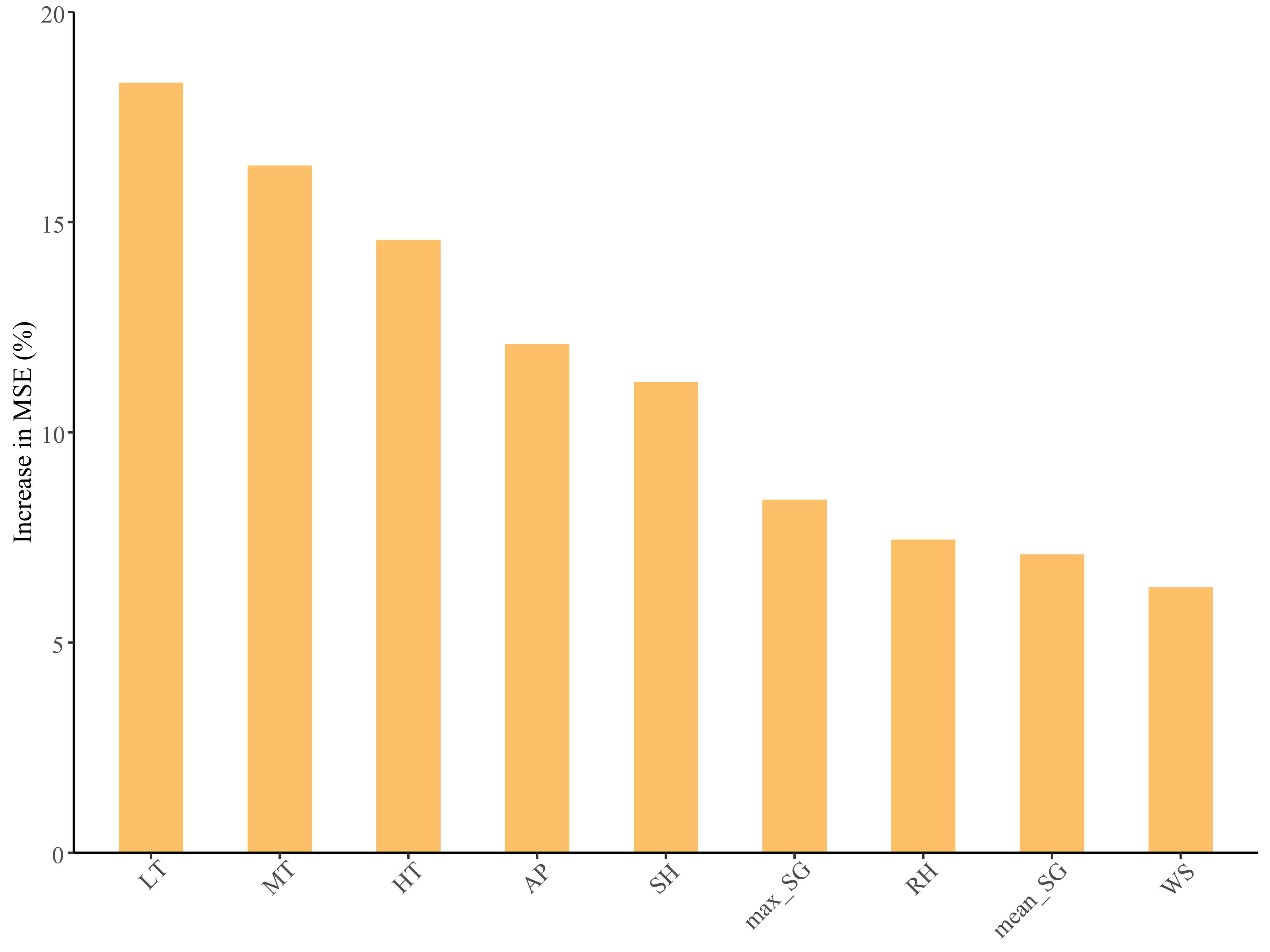

Supplement: S4 Fig — %IncMSE means the increase in the means of squared residuals. mean_SG: weekly mean speed of gustiness; max_SG: weekly mean maximum speed of gustiness; WS: weekly mean wind speed; AP: weekly mean air pressure; RH: weekly mean relative humidity; MT: weekly mean temperature; HT: weekly mean highest temperature; LT: weekly mean lowest temperature; SH: weekly mean sunshine duration. MSE: mean of squared residuals. (TIF) [file pntd.0012266.s004.tif]

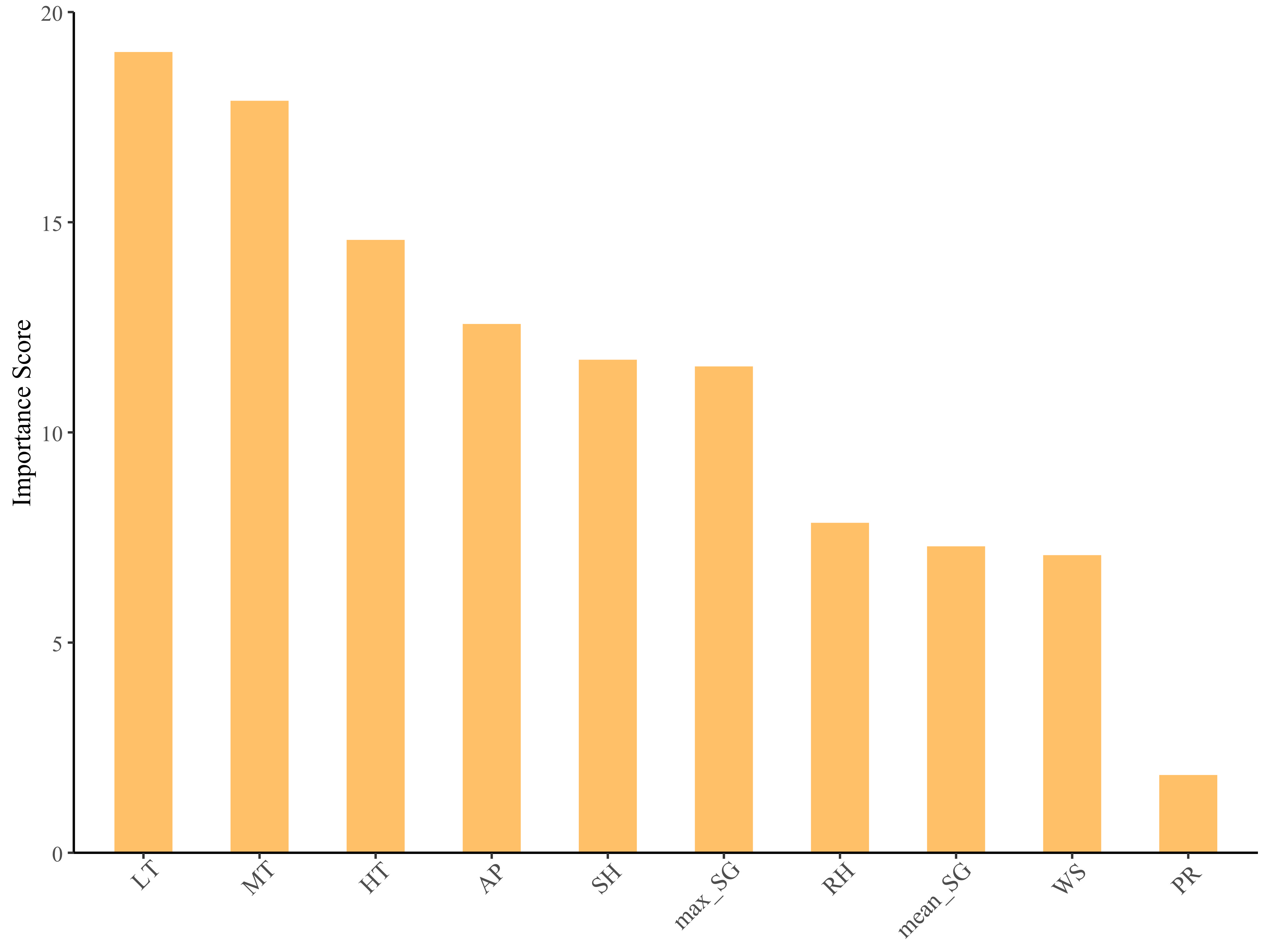

Supplement: S5 Fig — mean_SG: weekly mean speed of gustiness; max_SG: weekly mean maximum speed of gustiness; WS: weekly mean wind speed; AP: weekly mean air pressure; RH: weekly mean relative humidity; MT: weekly mean temperature; HT: weekly mean highest temperature; LT: weekly mean lowest temperature; PR: weekly mean precipitation; SH: weekly mean sunshine duration. (TIF) [file pntd.0012266.s005.tif]

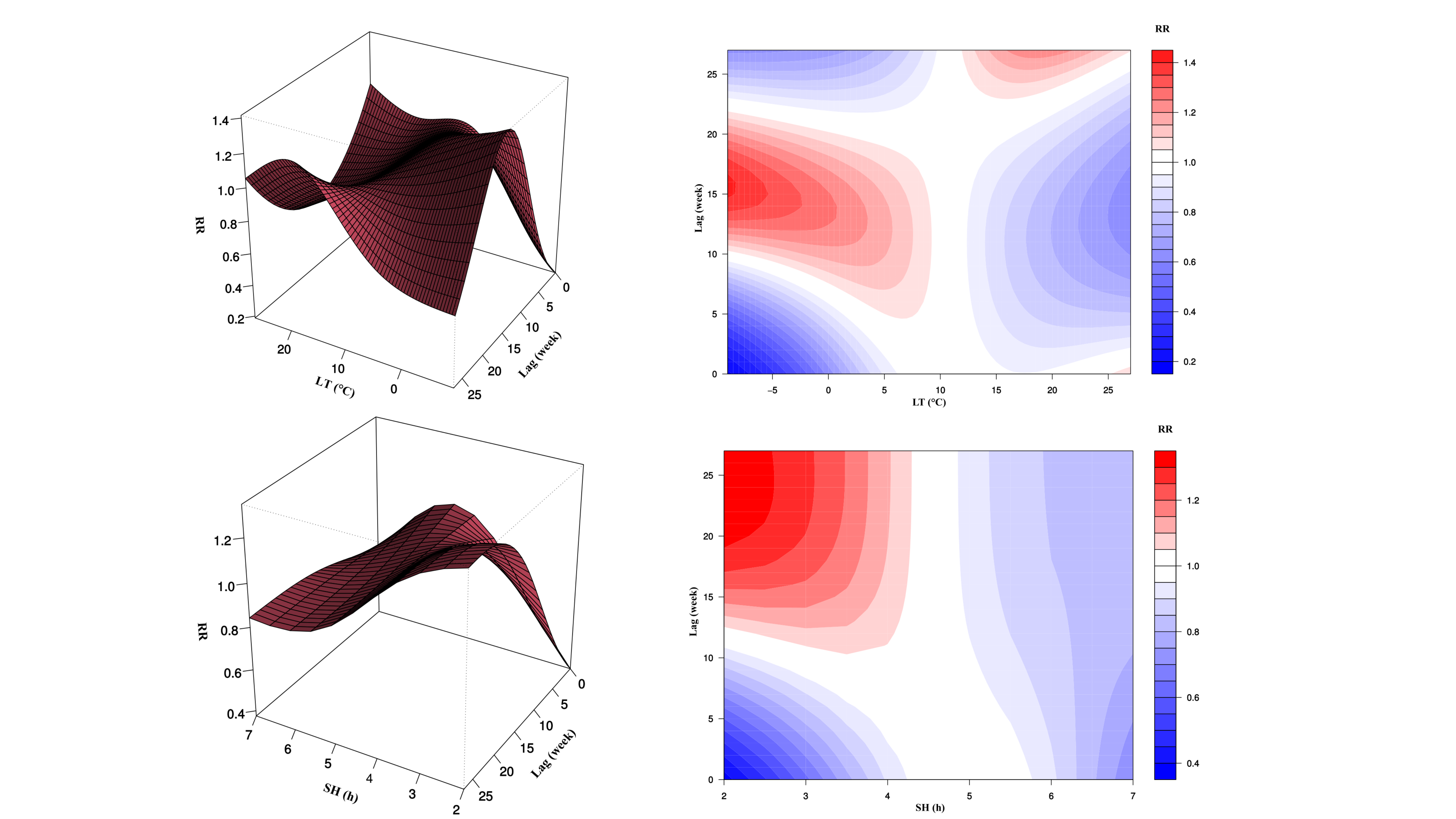

Supplement: S6 Fig — S3A and S3C Fig are 3-D graphs, S3B and S3D Fig are contour plots; S3A and S3B Fig show the effects corresponding to different combinations of lowest temperatures and lag weeks; S3C and S3D show Fig the effects corresponding to different combinations of sunshine durations and lag weeks; LT: weekly mean lowest temperature; SH: weekly mean sunshine duration. (TIF) [file pntd.0012266.s006.tif]

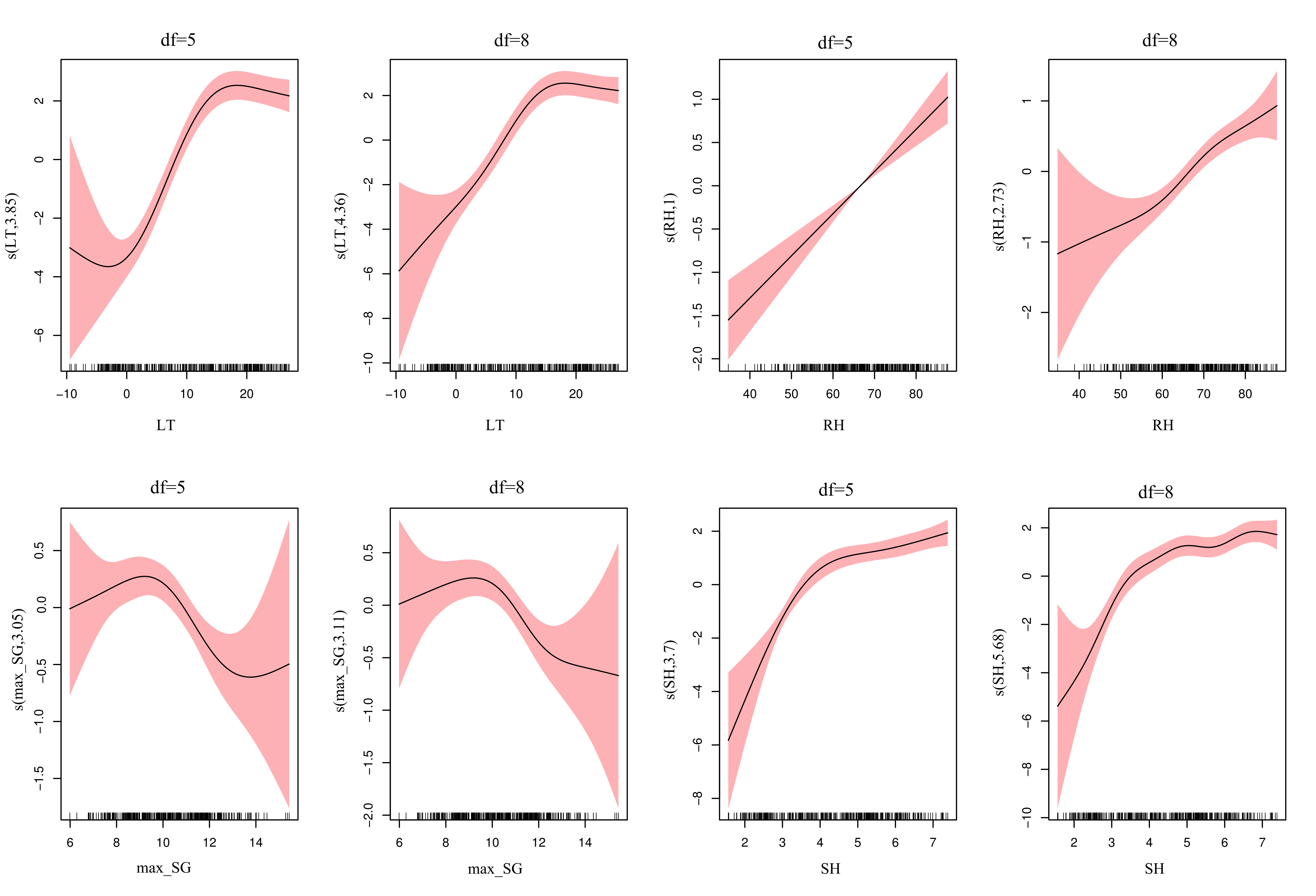

Supplement: S7 Fig — S4A and S4B Fig illustrate the exposure-curve relationship between lowest temperature and SFTS incidence for degrees of freedom 5 and 8, respectively; S4C and S4D Fig illustrate the exposure-curve relationship between relative humidity and SFTS incidence for degrees of freedom 5 and 8, respectively; S4E and S4F Fig illustrate the exposure-curve relationship between maximum speed of gustiness and SFTS incidence for degrees of freedom 5 and 8, respectively; S4G and S4H Fig illustrate the exposure-curve relationship between sunshine duration and SFTS incidence for degrees of freedom 5 and 8, respectively; LT: weekly mean lowest temperature; SH: weekly mean sunshine duration; max_SG: weekly mean maximum speed of gustiness; RH: weekly mean relative humidity. (TIF) [file pntd.0012266.s007.tif]

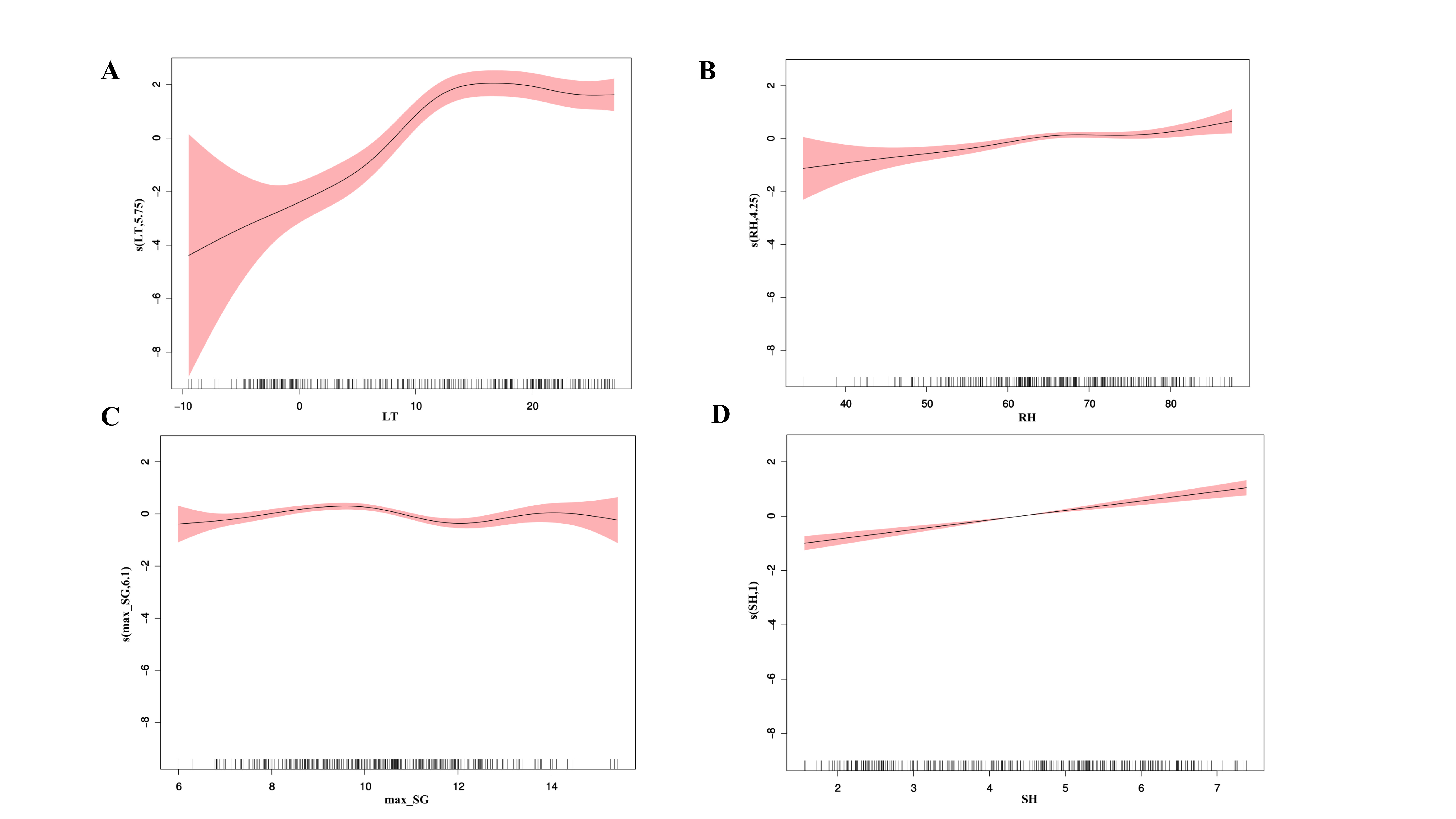

Supplement: S8 Fig — S5A Fig: adjusted for relative humidity, precipitation, wind speed and sunshine duration; S5B Fig: adjusted for lowest temperature, precipitation, wind speed and sunshine duration; S5C Fig: adjusted for lowest temperature, precipitation, relative humidity and sunshine duration; S5D Fig: adjusted for lowest temperature, precipitation, relative humidity and wind speed. LT: weekly mean lowest temperature; SH: weekly mean sunshine duration; max_SG: weekly mean maximum speed of gustiness; RH: weekly mean relative humidity. (TIF) [file pntd.0012266.s008.tif]

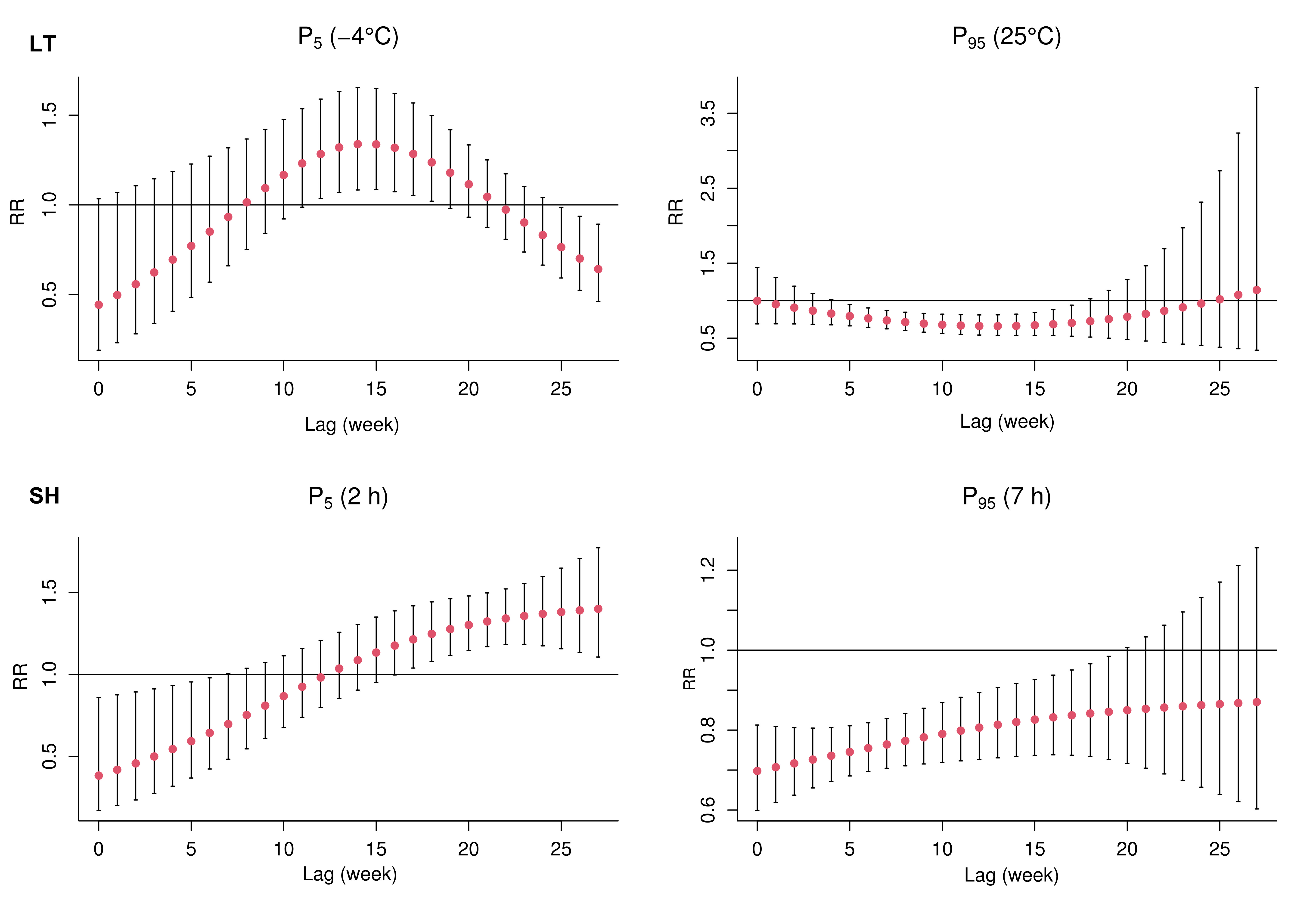

Supplement: S9 Fig — The upper-left and upper-right graph represent the effects of the 5th (-4°C) and 95th (25°C) percentiles of lowest temperature across different lag periods, compared to the median temperature, respectively; the lower-left and lower-right graph represent the effects of the 5th (2h) and 95th (7h) percentiles of sunshine duration across different lag periods, compared to the median sunshine duration, respectively. LT: weekly mean lowest temperature; SH: weekly mean sunshine duration. (TIF) [file pntd.0012266.s009.tif]

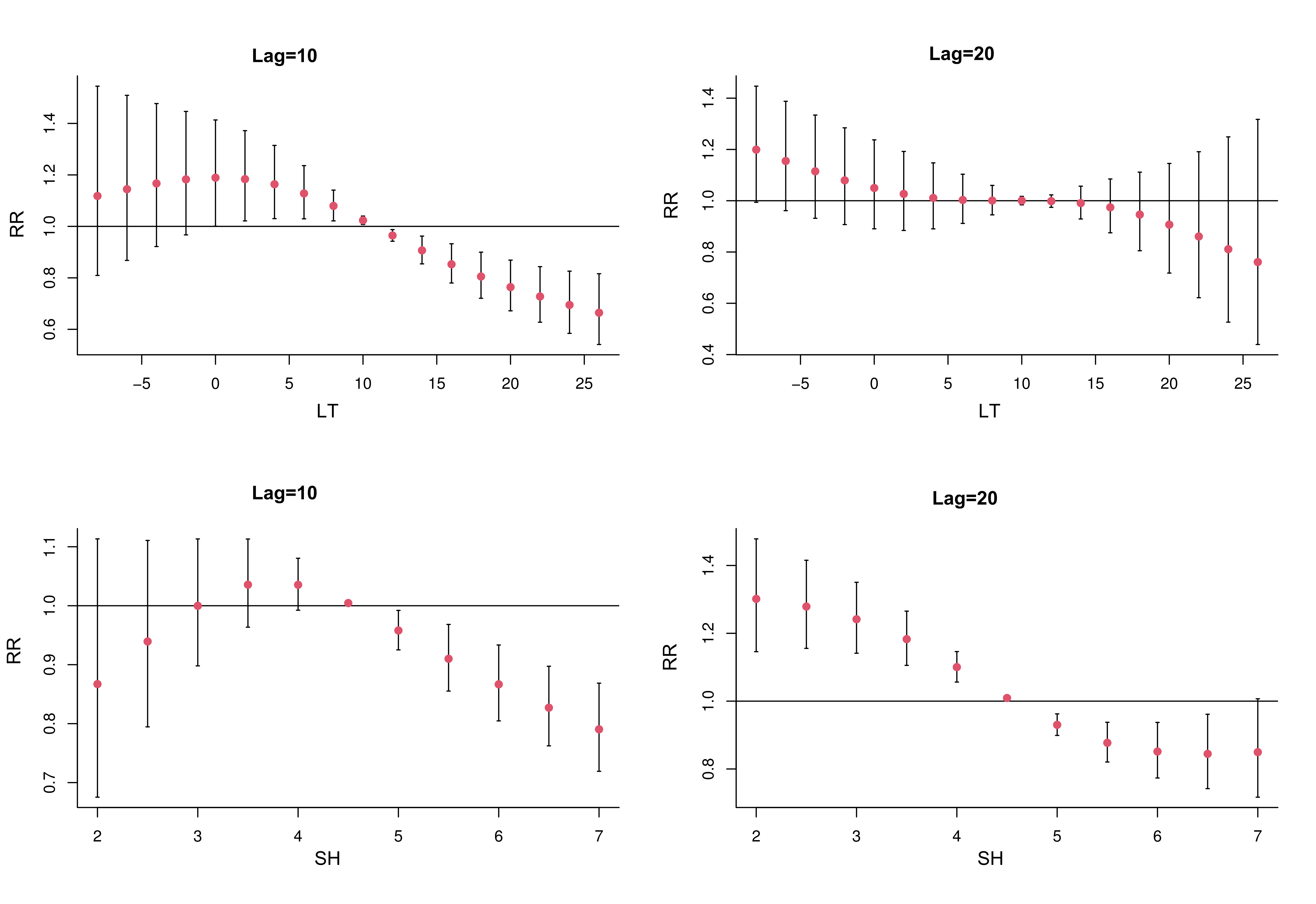

Supplement: S10 Fig — The upper-left and upper-right graph represent the effects of different minimum temperatures at the lag 10th and 20th weeks, respectively; the lower-left and lower-right graph represent the effects of different sunshine durations at the lag 10th and 20th weeks, respectively. LT: weekly mean lowest temperature; SH: weekly mean sunshine duration. (TIF) [file pntd.0012266.s010.tif]

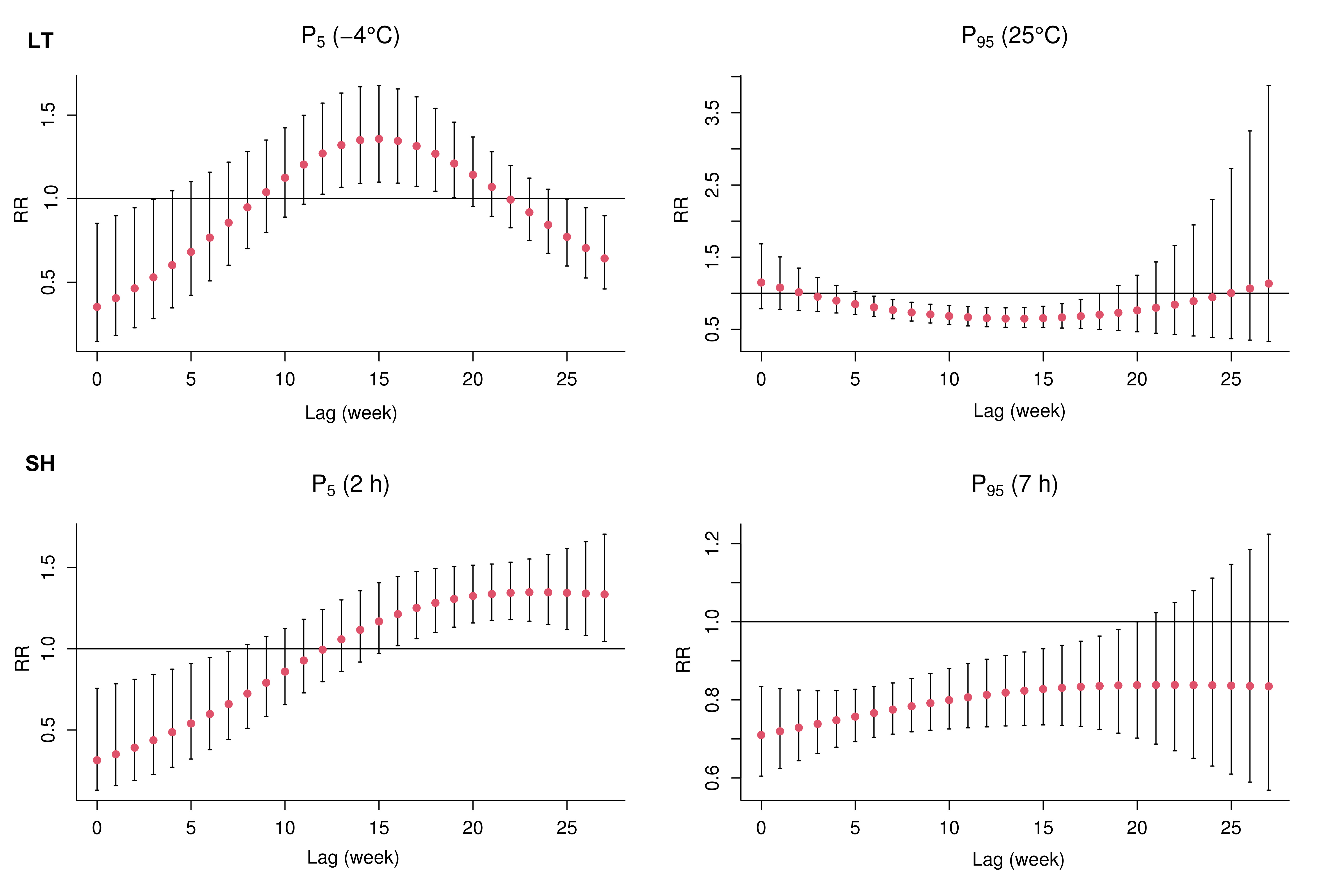

Supplement: S11 Fig — The upper-left and upper-right graph represent the effects of the 5th (-4°C) and 95th (25°C) percentiles of lowest temperature across different lag periods, compared to the median temperature, respectively; the lower-left and lower-right graph represent the effects of the 5th (2h) and 95th (7h) percentiles of sunshine duration across different lag periods, compared to the median sunshine duration, respectively. LT: weekly mean lowest temperature; SH: weekly mean sunshine duration. (TIF) [file pntd.0012266.s011.tif]

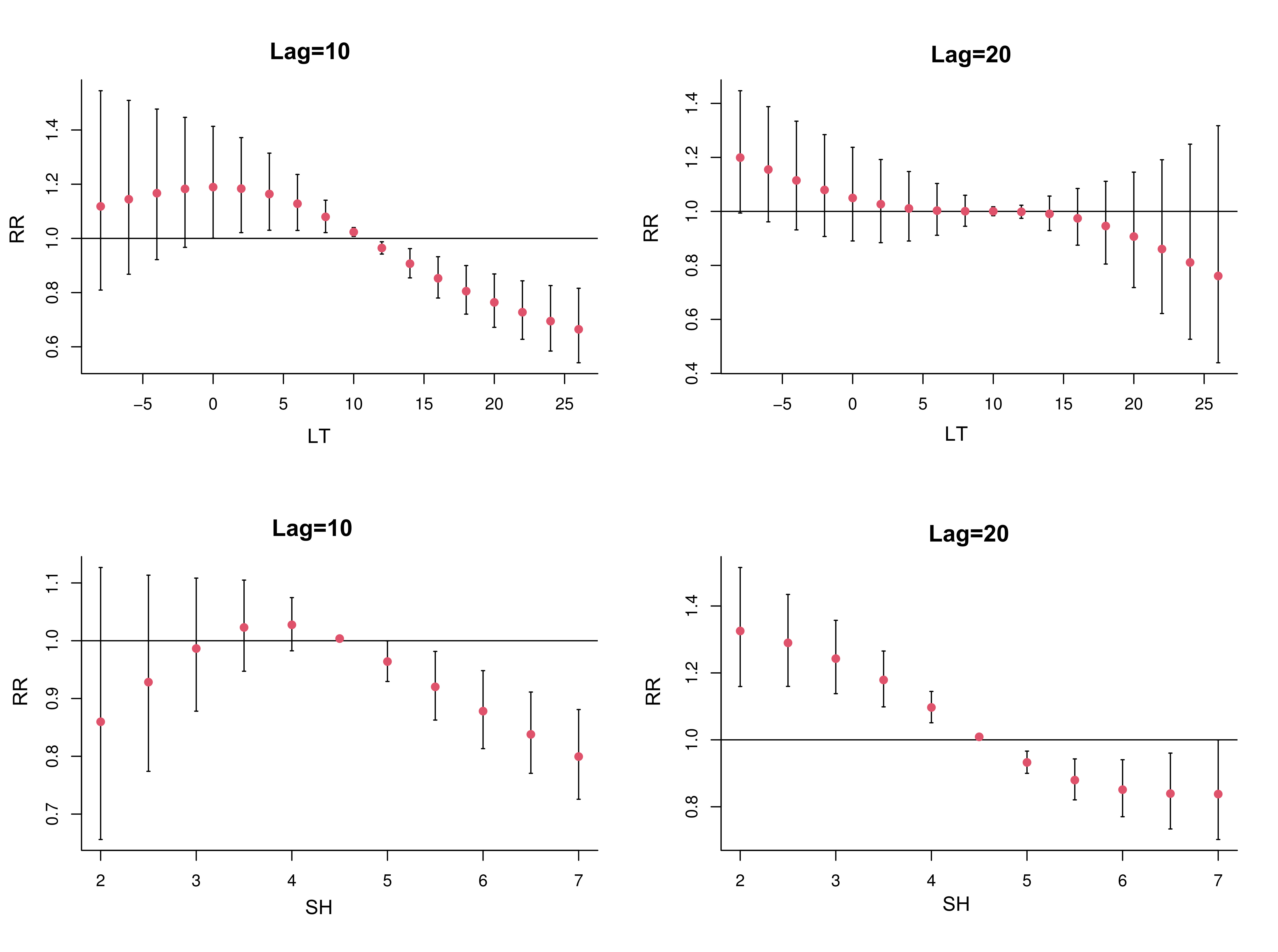

Supplement: S12 Fig — The upper-left and upper-right graph represent the effects of different minimum temperatures at the lag 10th and 20th weeks, respectively; the lower-left and lower-right graph represent the effects of different sunshine durations at the lag 10th and 20th weeks, respectively; LT: weekly mean lowest temperature; SH: weekly mean sunshine duration. (TIF) [file pntd.0012266.s012.tif]

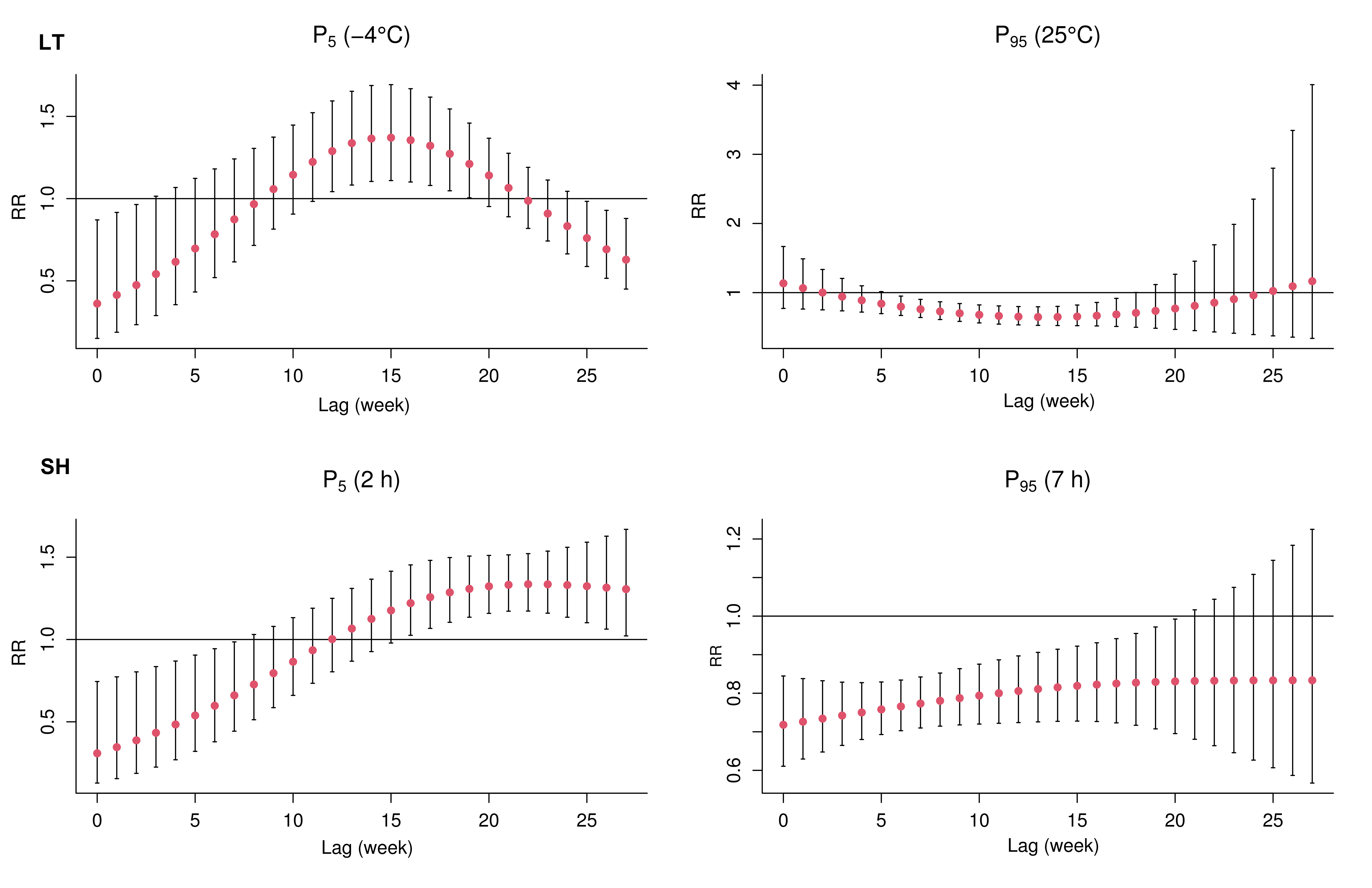

Supplement: S13 Fig — The upper-left and upper-right graph represent the effects of the 5th (-4°C) and 95th (25°C) percentiles of lowest temperature across different lag periods, compared to the median temperature, respectively; the lower-left and lower-right graph represent the effects of the 5th (2h) and 95th (7h) percentiles of sunshine duration across different lag periods, compared to the median sunshine duration, respectively; LT: weekly mean lowest temperature; SH: weekly mean sunshine duration. (TIF) [file pntd.0012266.s013.tif]

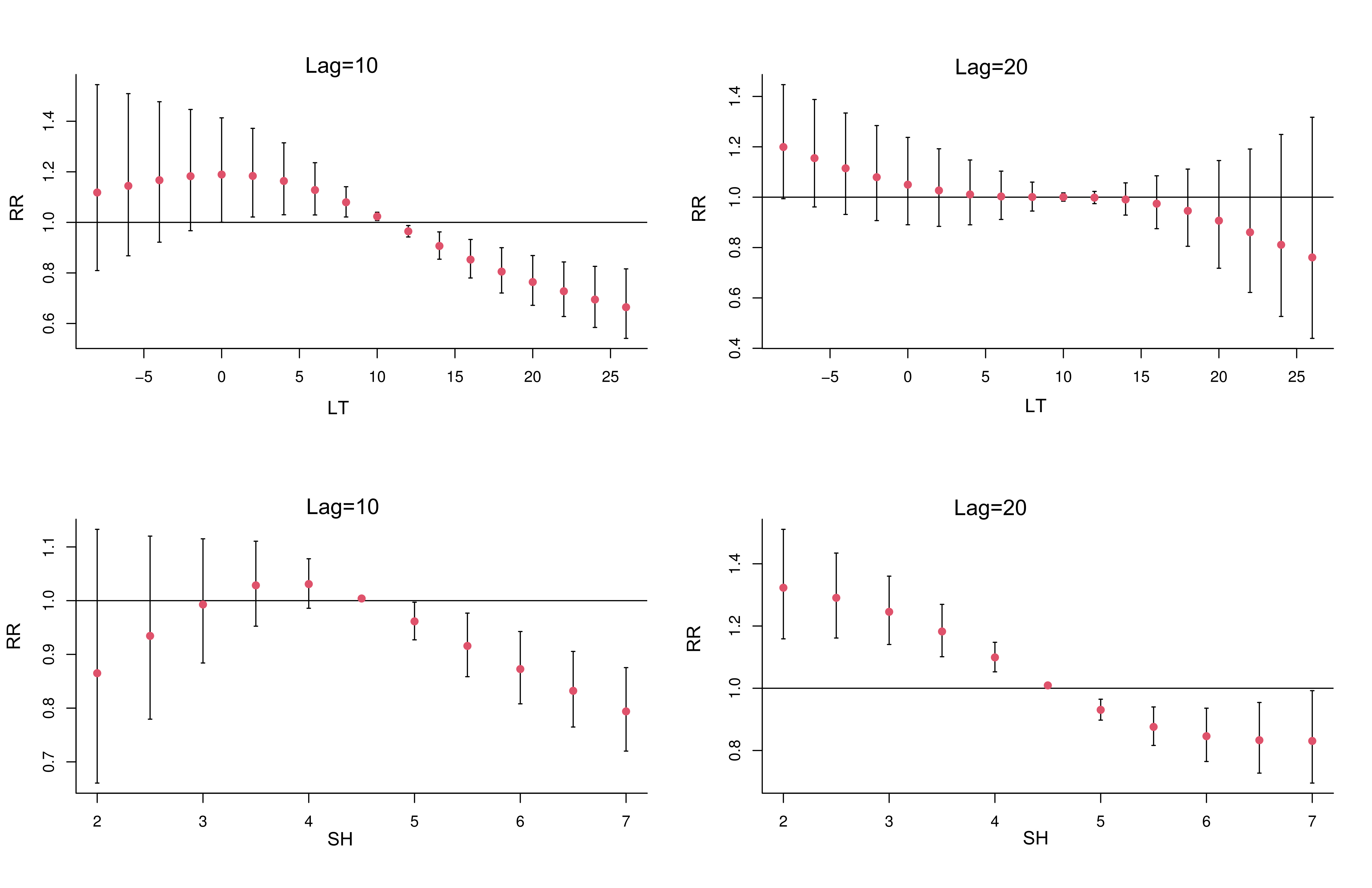

Supplement: S14 Fig — The upper-left and upper-right graph represent the effects of different minimum temperatures at the lag 10th and 20th weeks, respectively; the lower-left and lower-right graph represent the effects of different sunshine durations at the lag 10th and 20th weeks, respectively; LT: weekly mean lowest temperature; SH: weekly mean sunshine duration. (TIF) [file pntd.0012266.s014.tif]
